# Commercial Bio-Packaging to Preserve the Quality and Extend the Shelf-Life of Vegetables: The Case-Study of Pumpkin Samples Studied by a Multimethodological Approach

**DOI:** 10.3390/foods10102440

**Published:** 2021-10-14

**Authors:** Giacomo Di Matteo, Paola Di Matteo, Matteo Sambucci, Jacopo Tirillò, Anna Maria Giusti, Giuliana Vinci, Laura Gobbi, Sabrina Antonia Prencipe, Andrea Salvo, Cinzia Ingallina, Mattia Spano, Anatoly P. Sobolev, Noemi Proietti, Valeria Di Tullio, Paola Russo, Luisa Mannina, Marco Valente

**Affiliations:** 1 Dipartimento di Chimica e Tecnologie del Farmaco, Laboratorio di Chimica degli Alimenti, Sapienza Università di Roma, Piazzale Aldo Moro 5, 00182 Roma, Italy; giacomo.dimatteo@uniroma1.it (G.D.M.); andrea.salvo@uniroma1.it (A.S.); cinzia.ingallina@uniroma1.it (C.I.); mattia.spano@uniroma1.it (M.S.); 2 Dipartimento di Ingegneria Chimica Materiali Ambiente, Sapienza Università di Roma, Via Eudossiana 18, 00184 Roma, Italy; p.dimatteo@uniroma1.it (P.D.M.); matteo.sambucci@uniroma1.it (M.S.); jacopo.tirillo@uniroma1.it (J.T.); marco.valente@uniroma1.it (M.V.); 3 Dipartimento di Medicina Sperimentale, Sapienza Università di Roma, Viale Regina Elena 324, 00161 Roma, Italy; annamaria.giusti@uniroma1.it; 4 Dipartimento di Management, Sapienza Università di Roma, Via del Castro Laurenziano 9, 00161 Roma, Italy; giuliana.vinci@uniroma1.it (G.V.); laura.gobbi@uniroma1.it (L.G.); sabrinaantonia.prencipe@uniroma1.it (S.A.P.); 5 Laboratorio di Risonanza Magnetica “Segre-Capitani”, Istituto per i Sistemi Biologici, Area della Ricerca di Roma 1, CNR, Via Salaria Km 29.300, 00015 Monterotondo, Italy; anatoly.sobolev@cnr.it (A.P.S.); noemi.proietti@cnr.it (N.P.); valeria.ditullio@cnr.it (V.D.T.)

**Keywords:** pumpkin, biofilm, shelf life, metabolomics, NMR spectroscopy, NMR relaxometry, SPME-GC-MS analysis, biogenic amines, mechanical characterization, microbiological analysis

## Abstract

A multidisciplinary protocol is proposed to monitor the preservation of fresh pumpkin samples (FP) using three commercial polymeric films: A made of biodegradable cellophane from regenerated cellulose pulp; B from corn starch, cassava and eucalyptus, C made of polylactic acid from corn starch, and a polyethylene film used as reference (REF). Chemical, mechanical and microbiological analyses were applied on packaging and fresh and packaged samples at different times. After an 11-day period, NMR spectroscopy results showed a sucrose increase and a malic acid decrease in all the biofilms with respect to FP; fructose, glucose, galactose levels remained quite constant in biofilms B and C; the most abundant amino acids remained quite constant in biofilm A and decreased significantly in biofilm B. From microbiological analyses total microbial count was below the threshold value up to 7 days for samples in all the films, and 11 days for biofilm C. The lactic acid bacteria, and yeasts and molds counts were below the acceptability limit during the 11 days for all packages. In the case of biofilm C, the most promising packaging for microbiological point of view, aroma analysis was also carried out. In this paper, you can find all the analysis performed and all the values found.

## 1. Introduction

Food packaging plays an essential role in preserving food throughout the distribution chain. Without packaging, the direct contact with physical, chemical, and biological contaminants can drastically compromise food quality and nutritional health value. In this regard, food industries have increased attention towards products with improved nutritional quality and health promoting properties and towards the use of proper packaging materials. Packaging has to be capable of maintaining food sensorial and nutritional quality and guaranteeing food safety both in international and national markets even in complex conditions. In the twentieth century, the innovation of packaging underwent a rapid evolution thanks also to the discovery and marketing of new materials as plastics and derivatives. Nowadays, in many countries, including Italy, more than 70% of packaging is destined for the food industry. However, over time, the generation of a large amount of food packaging waste has become an extremely critical point, with Europe reaching 65% of the total packaging waste production. Traditional polymeric materials from mineral oils are characterized by long degradation times and are the main responsible for plastic pollution and environmental damage. In addition, this type of packaging can represent a possible carrier of food contaminants. The introduction of eco-sustainable bio packaging in food packaging represents a current exigency to which the scientific community and national and international legislative bodies have been working together.

In this paper, the mechanical and chemical/physical properties as well as the functionality of three commercial biofilms were investigated. The application of these biofilm to food packaging is limited (i.e., biofilm A is used in dairy, chocolate and confectionery; biofilm B is used for meat, while C for sandwiches, cookies, or cold treats to go), and they were not previously applied for fresh fruits and vegetables. 

The biofilm functionality on fresh-cut pumpkin sample preservation was examined. Pumpkins are generally large vegetables, and thus have difficulties in marketing, storage and handling, which leads to many losses. To reduce these problems minimally processed pumpkin is proposed as an alternative. However, this product is more perishable and needs conservation at refrigerated conditions and the use of a suitable packaging. Plastic packaging and modified atmosphere, but not biofilms, were investigated to this aim and their effect on physical and chemical properties of pumpkin were investigated [1,2,3]. Analyses of mass loss, microbiological growth, pH, aw, carotenoids content and sensory quality were carried out in order to obtain information on the shelf life of the minimally processed pumpkin [1,2,3]. 

The biofilm functionality was here examined monitoring the chemical profile of fresh and packaged products by means of untargeted and targeted methodologies. The untargeted NMR analysis was used to obtain a comprehensive metabolite profiling of pumpkin samples [4,5] whereas target analyses were focused on a specific class of compounds: total carotenoids, chlorophylls and polyphenols in pumpkins were determined by spectrophotometric methods; volatile compounds were determined by SPME-GC-MS. To evaluate the quality of fresh and packaged pumpkins, the presence of biogenic amines was monitored by means of HPLC analysis and microbiological analyses carried out. The potential changes in water dynamics during sample preservation were also investigated by time-domain NMR portable relaxometry. Finally, the antioxidant capacity of pumpkin extracts was also evaluated by standard assays.

## 2. Materials and Methods

### 2.1. Biodegradable Commercial Films

Commercial biodegradable films (biofilms) of vegetable origin, available on the market (here reported as A, B and C), and a commercial low-density polyethylene (LDPE) film used as a reference (REF), were used as food bags. The biofilm compositions as reported on the package label is reported in the Table 1. For polyethylene, which is a common packaging material (30 µm in thickness), oxygen transmission rate (OTR) and carbon dioxide transmission rate (CO_2_TR) are data available from the supplier (i.e., OTR 5396 +/− 420 cm^3^ m^−2^ day^−1^; CO_2_TR 26249 +/− 2918 cm^3^ m^−2^ day^−1^). 

### 2.2. Pumpkin and Packaged Food Preparation 

Pumpkins (*Cucurbita moschata cultivar*) were provided by a local farm (Tenuta di Dragone Corsetti, Rome, Italy). A variety of French origin, with medium-sized fruits of 8–15 kg that can be kept whole for more than 6 months after harvest. This pumpkin has a reddish–ocher skin, with evident grooves. The pulp, orange in color, is very thick and tasty.

**Figure 1:**
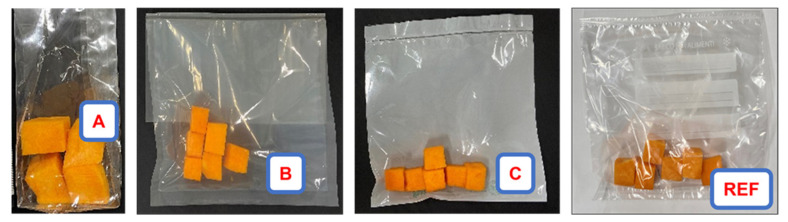
Pumpkin cubes packaged in the commercial biofilms (A, B, C) and polyethylene film (REF).

Pumpkins were harvested in October 2020, transported within 2 h directly from the field to the laboratory under refrigerated conditions and stored at 10°C before use. Pumpkins were washed with 2.5% sodium bicarbonate solution and then cut into cubes of about 2 cm side. The fresh pumpkin samples obtained at this stage (time zero) were reported as FP. FP cubes, about 100 g, were packaged using the three commercial biofilms (A, B, and C) and a polyethylene film (REF), see Table 1, each package having a volume of about 1000 cm^3^ (Figure 1). All the packaged samples were stored at 5 °C for 11 days. For NMR, total chlorophyll, carotenoid and polyphenol analyses and the measurements of the antioxidant capacity, samples were freeze-dried by Lyovapor^TM^ L-200 (Buchi, Milan, Italy) at 0.200 mbar for 12 h, blended and analyzed at 0 and 11 days.

For microbiological analyses, three samples for each package were randomly selected at 0, 4, 7 and 11 days.

### 2.3. Biofilms Analyses 

#### 2.3.1. Mechanical Characterization: Tensile Test

Tensile properties of the commercial biofilms were evaluated using a Z10 mechanical testing machine (Zwick-Roell, Ulm, Germany), in accordance with ISO 527-3 standard method [6]. The specimens were extracted from the films by a MT1130 die-cutter (MinEuro, Turin, Italy), using a metallic dog-bone-shaped cutting edge to obtain samples with a width of 4 mm and a gauge length of 30 mm. Tests were performed with a 200 N load cell, a crosshead speed of 5 mm/min, and a pre-load of 5 MPa. The tensile strain of the specimens was obtained by recording the crosshead displacement of the test frame. TestXpert software (Zwick-Roell, Ulm, Germany) was used to record the data. For each type of biofilm, at least 3 representative samples were investigated and the mechanical properties, in terms of tensile strength (σ_t_), elastic modulus (E), and elongation-at-break (ε_b_) were reported. Before the analysis, the thickness of each material was measured (three measuring points) with a centesimal micrometer (measuring range: 0–25 mm/graduation: 0.01 mm), see Table 1.

#### 2.3.2. Gas Permeability Testing: Oxygen Transmission Rate (OTR) and Carbon Dioxide Transmission Rate (CO_2_TR)

**Figure 2:**
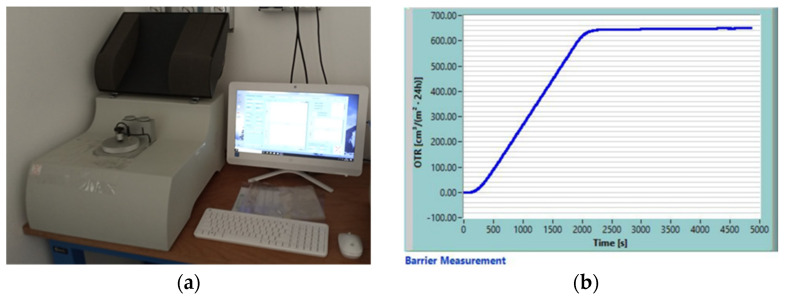
Set-up for gas permeability testing (**a**) and experimental “permeated gas volume-time” curve extrapolated from oxygen permeability test (**b**).

The oxygen transmission rate (OTR) and carbon dioxide transmission rate (CO_2_TR) were considered as indicators to evaluate the gas permeability performance of the commercial biofilms. The measurements were performed using a Perme permeability tester (ExtraSolution, Pisa, Italy) conforming to ISO 15105-2 (2003) standard method [7]. The testing apparatus can operate in a temperature range of 10–50 °C, relative humidity (RH) up to 95%, and with high-purity permeating gases (≥99.99% for CO_2_ and ≥99.95% for O_2_). The tests were performed at 23 °C, varying RH conditions in accordance with the type of measurement (50% RH for oxygen permeability test and 0% RH for carbon dioxide permeability test). The experimental set-up (Figure 2a) was based on gas/membrane/gas configuration. The film (sample surface of 50 cm^2^) was placed inside the measurement chamber separating the side of the testing gas from the side of the carrier gas (nitrogen). Pressure of the two sides was equal but testing gas partial pressure is different. Testing gas was transmitted through the film and carried to the sensor by nitrogen with a flow rate of 12.05 mL/min. Sensor measured the testing gases permeance in the carrier one, providing the transmission rate values. OTR and CO_2_TR, expressed in cm^3^ m^−2^ day^−1^, were extrapolated from the slope of the linear steady-state part of the permeated gas volume–time curve (Figure 2b). 

#### 2.3.3. NMR Analysis

For NMR analysis, Bligh–Dyer extracts were prepared according to the protocol previously described [8] with some modifications. Freeze dried sample (0.1 g) was sequentially added with a 3 mL methanol: chloroform (2:1 *v*/*v*) mixture and 0.8 mL of Millipore-grade water, shaking slightly after each addition. The obtained suspension was sonicated for 10 min in order to maximize the extraction yield. Then the extract was sequentially added with 1 mL of chloroform and 1 mL Millipore-grade water stirring with vortex for 30 s in order to achieve the phase separation. The emulsion was centrifugated at 7197× *g* for 15 min at 4 °C. The organic and hydroalcoholic phases were separated. To ensure the complete extraction of the soluble metabolites, the same procedure was repeated using pellet remained after the first extraction and half of the volumes of all the solvents. After phase separation a slow N_2_ flow at room temperature was used for drying. The obtained extracts were stored at −20 °C until analysis. The hydroalcoholic and organic extracts were analyzed using a Bruker AVANCE 600 spectrometer (Bruker, Milan, Italy) operating at the proton frequency 600.13 MHz and equipped with a Bruker multinuclear z-gradient 5 mm probe head. The NMR spectra were acquired at 28 °C according to the experimental conditions reported in most databases. Each dried hydroalcoholic extract was solubilized in 0.8 mL 400 mM phosphate buffer (pH 7.4)/D2O containing TSP 2 mM (internal standard) and EDTA-d16, filtered and transferred into a 5 mm NMR tube. ^1^H spectra of hydroalcoholic extracts were acquired using the zgpr sequence to suppress water signal by solvent presaturation. The NMR acquisition parameters were 256 transients, a recycle delay of 5 s, an acquisition time of 2.28 s, a 90° flip angle pulse of 15.65 µs, and 32 K data points. Each dried organic extract was solubilized in 0.8 mL of the CDCl3/CD3OD (2:1 *v*/*v*) mixture with tetramethylsylane (TMS) as internal standard, filtered and transferred into a 5 mm NMR tube that was flame sealed. ^1^H spectra (Bruker pulse sequence zg) of the organic extracts were acquired with 256 transients, a recycle delay of 5 s, an acquisition time of 1.82 s, a 90° flip angle pulse of 10.4 µs, and 32 K data points. Two-dimensional (2D) NMR experiments (^1^H-^1^H TOtal Correlated SpectroscopY (TOCSY), ^1^H-^13^C Heteronuclear single quantum coherence (HSQC), ^1^H-^13^C heteronuclear multiple bond correlation (HMBC), and ^1^H homonuclear J-resolved experiment (JRES), were performed on each hydroalcoholic and organic extract under the experimental conditions previously reported [9]. In order to evaluate the repeatability of the protocol, the entire procedure (from the sample preparation to NMR analysis) was carried out in duplicate. The identified signals in the hydroalcoholic extracts were integrated by the TOPSPIN (Version 4.1.1) software (Bruker, Milan, Italy) and normalized with respect to the methyl group integral of TSP signal (at 0.00 ppm) set to 100 (Table S1) in order to obtain the metabolite absolute quantities [5]. The metabolite signals used for the quantification were showed in Table 2. The metabolites’ amounts are expressed in mg/10 g of dried sample ± SD (standard deviation) (Figures S1 and S2).

The integral areas of 4 selected signals in the ^1^H NMR spectra of organic extracts (Figure S3) were measured using the TOPSPIN software and normalized with respect to the integral (I_FA_) of the -CH_2_ group signal of all fatty acids set to 100. The TMS signal was a chemical shift reference (0.00 ppm). The molar % values ± SD of fatty acids were calculated with consideration of the number of equivalent protons using the following equations:

%_TUFA_ = 100(0.5I_TUFA_/I_FA_) (1)

%_DUFA_ = 100(I_DUFA_/I_FA_) (2)

%_MUFA_ = 100(I_UFA_ − 2I_DUFA_ − 1.5I_TUFA_)/I_FA_ (3)

%_SFA_ = 100 − %_MUFA_ − %_DUFA_ − %_TUFA_ (4)

where %_TUFA_, %_DUFA_, %_MUFA_ and %_SFA_ are the molar % of tri-unsaturated fatty acids, di-unsaturated fatty acids, mono-unsaturated fatty acids, saturated fatty acids, respectively. I_TUFA_, I_DUFA_, I_UFA_ and I_FA_ are integrals. The amount of each quantified fatty acid in the organic extracts is reported in Table S2.

One-way analysis of variance (ANOVA) was realized by the Statistica software (Version 14.0.0.15) in order to determine significant differences (*p* < 0.05) between the results (Tables S3 and S4). 

#### 2.3.4. Spectrophotometric Analysis of Chlorophylls, Total Carotenoids, Total Polyphenols and Antioxidant Activity

Pigments and total polyphenols were analyzed both in fresh and packaged samples at different times: time 0 (fresh, t_0_) and after 11 days of storage (t_11_) at T = 5 ± 1 °C. Chlorophylls a and b extraction was performed according to a protocol previously described [10] with some modifications. A Folch extraction was carried out on 10 mg of lyophilized sample adding 6 mL of a chloroform: methanol (2:1 *v*/*v*) mixture, together with 10 mg of MgO in order to neutralize plant acids and avoid pheophytin formation [11]. The obtained suspension was sonicated for 10 min to maximize the extraction yield. The pellet was removed by filtration through a filter paper under vacuum condition. Then 1.2 mL of Millipore-grade water was added to achieve the phase separation by centrifugation at 7197 g for 5 min at 20 °C. The lower organic phase was dried by N_2_ flow and then solubilized in chloroform. The absorbance values at 648, 666 and 750 nm at 25 °C was detected by an UV/VIS spectrophotometer. The contents of chlorophyll a (Chl a), chlorophyll b (Chl b) were calculated according to Wellburn equations [12]. Data were reported as means of two replications and expressed as mg/g ± SD (standard deviation). Statistical significance was reached at *p* < 0.05.

Total carotenoid content was evaluated in the lipophilic fraction by UV-Vis methods at 449 nm, according to Rapa et al. [13] Briefly, 20 mL of ethanol:hexane mixture (4:3 *v*/*v*) was added to 0.015 g homogenized sample in a glass tube (protected from light). The carotenoids extraction was conducted by agitating the mixture for 1 h (darkness) at 200 rpm. Thereafter, 1 mL of distilled water was added to the mixture and stirred by inversion. The hexane fraction was then collected in an amber vial. The results were compared with a standard solution of β-carotene in n-hexane, and the quantification of total carotenoids was achieved by the linear regression (R^2^ = 0.9967) and expressed as milligram of β-carotene (mg BCE).

Total phenolic content was determined by spectrophotometric analysis according to a previously published method [14]. Briefly, 0.08 g of lyophilized pumpkin was extracted with 20 mL of methanol in aqueous solution (60:40, *v*/*v*). Samples were homogenized in an Ultra-Turrax for 3 min and centrifuged at 3000× *g* for 5 min. The total content of polyphenols was expressed as milligrams of gallic acid equivalent (GAE) per kg. The final results were obtained through a calibration curve ranging from 10 to 100 mg/L (R^2^ = 0.9997). 

Antioxidant activities were determined by DPPH and ABTS assays, according to the methods of Preti et al. [15]. The DPPH and ABTS∙ free radical scavenging activity of the pumpkin extracts was evaluated by measuring the decrease in absorbance at 515 nm (DPPH), and 734 nm (ABTS). The absorbance was measured in 1-cm path length cuvettes against methanol in aqueous solution (60:40, *v*/*v*), using a UV-Vis spectrophotometer (Jenway, Stone, UK). Results were expressed as inhibition rate and were calculated based on Equation (5):

(5) $$I\%=\frac{A_{0}-A_{f}\;}{A_{0}}\times 100$$

where *A*_0_ is the radical cation’s initial absorbance, and *A_f_* is the absorbance after the addition of sample extract.

#### 2.3.5. Biogenic Amines (BAs) Determination

The BAs determination was carried out according to a previously optimized method with some modifications [14]. About 0.4 g of lyophilized pumpkin sample was extracted with 10 mL of 0.6 M HClO_4_, homogenized for 3 min, and centrifuged at 2700× *g* for 10 min. The supernatant was filtered through a 0.45 μm membrane millipore filter and collected in a flask. The residue was added with 10 mL of 0.6 M HClO_4_, mixed, and again centrifuged for 10 min. Then, the second extract was then filtered and added to the first one. The final volume was adjusted to 25 mL with 0.6 M HClO_4_. An aliquot of 1 mL of the final extract was then derivatized by adding 200 μL of 2 M NaOH, 300 μL of saturated NaHCO_3_ solution, and 2 mL of dansyl chloride solution (10 mg mL^−1^ in acetone). After shaking, the samples were left in the dark for 60 min at 45 °C. About 100 μL of 25% NH_4_OH was added to stop the derivatizing reaction. The final volume was adjusted to 5 mL by adding acetonitrile. The dansylated extract was filtered using 0.45-μm filter (Whatman^®^ Puradisc filters, Sigma Aldrich, Milan, Italy), injected into the HPLC system, and analyzed with a previous standard method [15]. 

#### 2.3.6. Microbiological Analysis

The microbiological analysis was performed on a sample (about 25 g) aseptically removed from the package, placed in a stomacher bag, diluted with 0.9% NaCl solution and homogenized with a Stomacher LAB Blender 400 (Pbi International). Further dilutions were carried out using the same diluent. The total aerobic count was determined on 3M Petrifilm aerobic total count, with incubation at 30 °C for 24–48 h. Yeasts and molds were detected on 3 M Petrifilm yeast and molds at 30 °C for 24–72 h and Lactic acid bacteria were enumerated on 3M Petrifilm lactic acid bacteria incubated at 30 °C for 24–72 h. Measurements were performed on 3 samples for each package randomly selected, at 0, 4, 7 and 11 days. 

#### 2.3.7. Mass Loss Measurement

The percentage mass loss was determined according to the following Equation (6):

(6) $$\%\;M_{LOSS}\left(t\right)=\frac{M_{0}-M_{t}}{M_{0}}\cdot 100$$

where: % *M_LOSS_* (*t*) is the percentage mass loss at time t, *M*_0_ is the initial sample mass and *M_t_* is the sample mass at time *t*. The sample mass was determined by a digital precision balance (±0.1 g) (Gibertini, Novate, Italy). The mass loss was measured in triplicate: three samples for each package were randomly selected, at 4, 7 and 11 days. 

#### 2.3.8. Water Activity Measurement

Water activity was measured by means of a Humimeter (Schaller Humimeter Rh2). Measurements were performed in triplicate at 0, 4, 7 and 11 days. The samples and the instrument were adjusted to the surrounding temperature for at least 30 min. The screw top jar of the device was filled with two pieces of pumpkin (50.00 ± 1.00 g) and the water activity measuring chamber was closed. The measure was carried out after 10 min. 

#### 2.3.9. pH Evaluation

The pH was evaluated on the homogenized pumpkin by a pH meter (Crison GLP 21 Crison Instruments). The pH measurement was carried in triplicate at 0, 4, 7 and 11 days. 

#### 2.3.10. Carbon Dioxide Measurement

CO_2_ concentration in packaged pumpkin was measured using a Uniphos precision air sampling pump and gas detection tubes with different ranges of concentrations (0.03–0.5, 0.25–3.00 and 0.5–10% vol). For gas analysis, a volume of 100 cm^3^ of package headspace was taken. To avoid modifications in the headspace gas composition due to gas sampling, each package was used only for a single measurement of the headspace gas composition. Three bags were used for each measurement at 0, 4, 7 and 11 days. 

#### 2.3.11. Aroma Compounds by SPME-GC-MS Analysis

The aroma profile of pumpkin packaged in C film at initial time and at 11 days of storage at 5 °C was evaluated by SPME-GC-MS. 10 mL pumpkin pulps and 3.0 g of NaCl were added into 15 mL glass vial. The sample was equilibrated 40 min by oscillation at 45 °C [16]. A 100 μm polydimethylsiloxane fused silica coated fiber (Supelco Co., Bellefonte, PA, USA) was used for SPME. A micro-syringe was inserted into the headspace samples for 40 min at 45 °C. The desorption was carried out at 250 °C for 3.5 min in the injection port of an Agilent GC–MS (7890B–5977B). An Agilent Technologies, Inc., HP-5MS column was used (30 m, 0.25 mm I.D., 0.25 μm film thickness). The injection port was controlled in spitless type. The oven temperature program was from 45 to 140 °C at a rate of 5 °C/min, followed by an increase to 220 °C, at a rate of 10 °C/min; holding time was 5 min and finally the temperature was increased to 250 °C, and kept for 2 min in isothermal condition. The carrier gas was He at a constant flow rate of 1.0 mL/min. The MS fragmentation was carried through electronic effect EI at 70 eV, a source temperature of 230 °C and scan mode was between 50 and 550 mass units. 

#### 2.3.12. Portable Time Domain (TD)-NMR Relaxometry

Longitudinal and transverse relaxation times were measured at 13.62 MHz with a portable NMR instrument from Bruker Biospin interfaced with a purposely built single-sided sensor by RWTH Aachen University, Germany [17]. Longitudinal relaxation times T_1_ were measured with the aperiodic saturation recovery sequence, using a logarithmic increment, 64 blocks were collected in each experiment [18]. Effective transverse relaxation times T_2eff_ were measured using the CPMG pulse sequence with TE of 80 µs, and 4096 echoes. Because the field generated by unilateral NMR is strongly inhomogeneous (G = 14.28 T/m), transverse relaxation times obtained are shorter than those measured in a homogeneous field. The shortening of the transverse relaxation time can be minimized by using an echo spacing as short as possible. It should be noted that, using the CPMG sequence for measuring T_2_, only water components are observed, whereas T_2_ of the rigid macromolecular matrix which is of the order of a few tens of µs is neglected.

T_1_ and T_2_ were collected on four pumpkin samples packaged in biofilm B and biofilm C. Measurements were carried out to a depth of 2 mm inside the pumpkin sample and collected for 1, 4, 7, and 11 days. T_1_ data were obtained fitting the magnetization with the following equation:

(7) $$M_{z}\left(t\right)=M_{ze}\left(1-e^{-\frac{2t}{T_{1}}}\;\right)$$

where $M_{ze}$ is the magnetization at the equilibrium.

The transverse relaxation times T_2_ were obtained fitting the magnetization decay with the following equation:

(8) $$M_{xy}\left(t\right)=\sum_{i\;i=1}^{N}M_{0i\;\;}(e^{-\frac{2t}{T_{2i}}}\;)$$

where T_2i_ is the ith time constant of magnetization decay, $M_{xy}\left(t\right)$ is the component of magnetization on the *xy* plane and *M*_0_ is the initial magnetization for each ith T_2_ component. The proton density was calculated applying Equation (2) and extrapolating at zero time the total $M_{0t\;\;}$ value.

## 3. Results and Discussion

### 3.1. Mechanical and Chemical/Physical Characterization of the Commercial Biofilm 

It is important to notice that the investigated commercial eco sustainable films had a different but very uniform thickness, see Table 1. The different mechanical performances of the three commercial samples are primarily attributable to the nature of the raw materials employed for their production. 

#### 3.1.1. Mechanical Characterization: Tensile Test 

**Figure 3:**
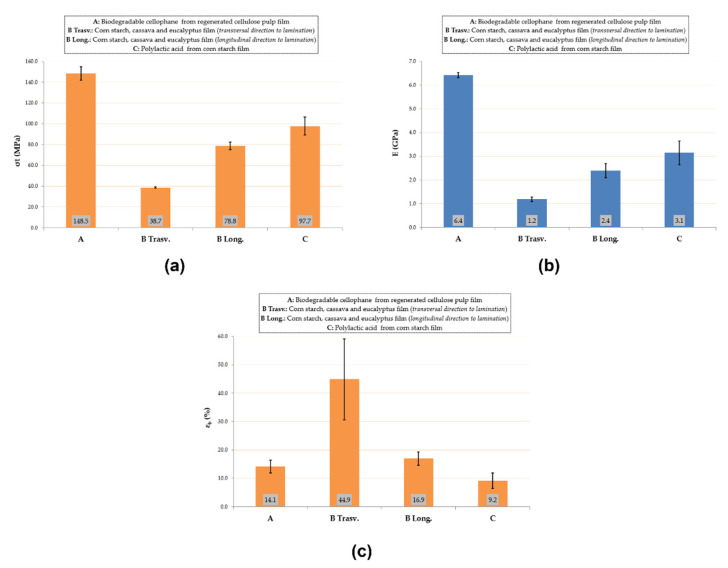
Tensile test results: tensile strength (**a**), elastic modulus (**b**), and elongation-at-break (**c**).

**Figure 4:**
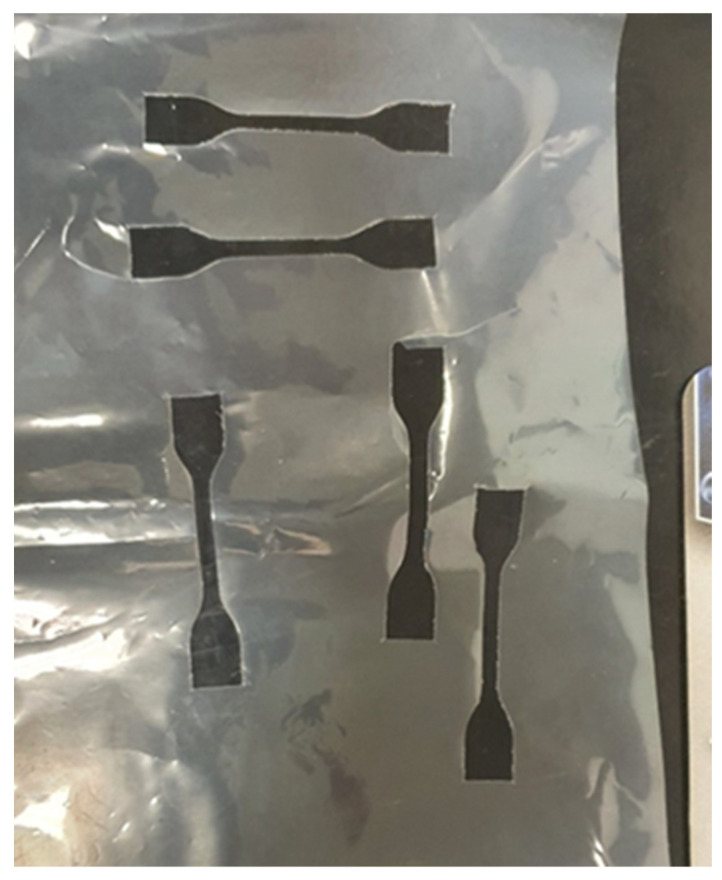
Cutting on B (Corn starch, cassava, and eucalyptus) biopolymer film to evaluate the mechanical properties with respect to the lamination direction.

Tensile test results are shown in Figure 3 The maximum σ_t_ can be found in A sample, since the cellulose-based formulation confers a higher strength and more stiff behavior than the other biofilms (Figure 3). In this regard, due to their mechanical properties, cellulose fillers are often employed as strengthening agents in packaging film blends [19]. Moreover, as clear from the histograms refer to B samples, the tensile response of the eco-films is strongly correlated to the cutting direction (Figure 3). Figure 4 highlights the cuts performed on B film to mechanically test the sample in the two orientations with respect to the extrusion direction. The specimens extracted in the direction transversal to the film extrusion (labeled with “trasv”) showed lower mechanical strength but significantly greater deformability than the specimens extracted in the longitudinal direction (labeled with “long”), resulting in an ε_b_-increase of 165% (calculated as a percentage increment with respect ε_b_-value found in B Long). Similar anisotropic behavior was demonstrated by Siracusa et al. [20] on C films for food packaging applications. Regardless of the specimen configurations, B film showed more ductile and deformable behavior than the other films, indicating better mechanical flexibility requirements for packaging applications, where it is crucial to avoid breaking during processing and use [21]. 

#### 3.1.2. Gas Permeability Testing: Oxygen Transmission Rate (OTR) and Carbon Dioxide Transmission Rate (CO_2_TR)

The gas permeability of plastic films is of primary importance in food packaging. The ability to preserve the contents of the package from contact with the air is essential to avoid degradation phenomena, ensuring a correct and healthy shelf life of the products. Furthermore, the barrier property of a packaging film is crucial in the context of modified atmosphere packaging (MAP) technology: packaging systems that involve changing the gaseous atmosphere surrounding a food product inside a pack and employing packaging materials with an appropriate permeability to maintain the changed atmosphere at an acceptable level for preservation [22]. In this framework, the oxygen barrier performances of a film are fundamental because oxygen promotes a lot of degradation mechanisms of food, including corrosive phenomena, oxidation, and great modification of organoleptic properties. In addition, carbon dioxide plays a key role for MAP systems because it can potentially reduce the degradation phenomena associated with processed fresh vegetable products, leading to a significantly longer shelf-life [23]. Table 1 reports the experimental values of OTR and CO_2_TR for the three films under examination. 

Concerning the oxygen permeability performance, only A and B films provided proper requirements for food packaging applications as reported in [24], which defines recommended OTR value lower than 20 cm^3^ m^−2^ day^−1^. The suitability of these films for the food packaging sector is also demonstrated in terms of carbon dioxide permeability, considering that the recommended CO_2_TR index should range from 3 to 5 times the OTR value [25]. As verified by Piscopo et al. [26], the higher CO_2_TRs values than OTR ones are to be attributed to the different behavior of carbon dioxide molecules in terms of solubility and diffusion through the polymeric chains, although the molecular size is larger. 

The experimental results are in good agreement with the literature data. For A film, OTR values between 3.4 cm^3^ m^−2^ day^−1^ and 11.1 cm^3^ m^−2^ day^−1^ were detected [27]. Corn starch-based packaging materials (such as B sample investigated in this work) show an OTR value close to 1 cm^3^ m^−2^ day^−1^ [28]. Discrepancies between the experimental and literature/datasheet values are attributable to the different standard methods and test conditions (e.g., temperature and RH) involved in the analysis of the films. For PLA-based film under study, no reference values are available. However, analogous commercial films tested in other research works reported similar results: OTR of 487.67 cm^3^ m^−2^ day^−1^ and CO_2_TR of 1201 cm^3^ m^−2^ day^−1^ reported in [20]; OTR of 617.6 cm^3^ m^−2^ day^−1^ reported in [27]. Considering these values and the recommended level for packaging applications, C material would seem not suitable for medium or long shelf-life of some food products. This evidence finds agreement with previous gas permeability investigations on PLA bio packages [29,30], which stated inferior barrier properties with respect to conventional petroleum-based polymers. 

### 3.2. Chemical Profile of Fresh and Packaged Samples 

#### 3.2.1. NMR Based Metabolite Profile 

The metabolite profile of pumpkin samples, as obtained by the NMR methodology, is reported here for the first time. The assignment of ^1^H NMR spectra of Bligh–Dyer hydroalcoholic extracts was carried out using two-dimensional NMR experiments (^1^H-^1^H TOCSY, ^1^H-^13^C HSQC, ^1^H-^13^ HMBC and JRES experiments) and literature data regarding other vegetable matrices [31,32]. Sixteen free amino acids, five sugars, four organic acids and choline and trigonelline were identified and quantified (Table 3). In particular, pyroglutamate, generally identified only in vegetables, was identified by the typical doublet of doublets signal at 4.1 ppm (J = 5.9 Hz; 9.1 Hz) due to α-CH that showed cross peaks correlations with β-CH2, β’-CH2 and γ-CH2 at 2.04, 2.51 and 2.41 ppm, respectively, observed in the ^1^H-^1^H TOCSY map [33,34]. The most abundant sugars, namely sucrose, glucose, and fructose were identified taking into account their different isomeric form present in solution. Additionally, less abundant galactose and myo-inositol were also identified.

The assignment of ^1^H NMR spectra of Bligh–Dyer organic extract was also carried out reporting the main classes of fatty acids (Table 4).

In order to establish the functionality of the three commercial biofilms, the metabolite profile of packed pumpkins after 11 days was compared to that of fresh samples FPs (time zero). Regarding sugars, the sucrose level increased for all the examined biofilms compared to FP, in particular for biofilm A it was 1.5 times higher. The fructose, glucose and galactose amounts were quite constant for all the tested biofilms, except for bio-film A package that showed a significant decrease of these metabolites. Myo-inositol content was quite steady for all the biofilms. Regarding organic acids, the malic acid amount decreased significantly for all the selected biofilms compared to FP, in particular, for biofilm REF it was more than 1.5-fold lower. On the contrary, an increment of citric acid was observed for both biofilms A and C (at least 1.5-fold), and those of fumaric acid for both biofilms B and REF (at least 1.4-fold). Formic acid remained quite constant for all the bio-film packages. Regarding amino acids, the most abundant amino acids in pumpkins (aspartate, glutamine, asparagine, arginine and pyro-glutamate) decrease in biofilm B packaged vegetable respect to FP, in particular pyro-glutamate was 1.5-fold lower. A decrease in asparagine was also observed for biofilm C and those of arginine for both bio-films C and REF. The tyrosine amount significantly decreased in the case of three commercial biofilms, in particular in biofilm B package it was 1.5-fold lower. Both alanine and histidine levels varied a lot for all the tested commercial packaging solutions. In particular, the alanine content decreased for bio-film A and increased more than 3 and 2 times for biofilms B and C, respectively, whereas the histidine amounts showed an opposite trend with an increment in biofilm A packed vegetable and a decrease in both biofilms B and C packed pumpkin respect to FP. Biofilm C package showed an increment of glutamate (1.5-fold) and a decrease of isoleucine, whereas biofilm B showed a decrease of both phenylalanine and tryptophan. All the other amino acids were quite steady for all the examined packaging’s, except for the lowest one (leucine) that decreased significantly for both biofilms C and REF. The trigonelline level increased significantly for biofilm C and decreased for both biofilms B and REF, and the choline amount decreased for both bio-films A and B compared with FP. Regarding the quantified fatty acids in the organic fraction, TOT SFA, TOT UFA and MUFA remained quite steady for all the tested biofilms. TUFA showed an increment for all the selected packaging, whereas DUFA increased for biofilm B and decreased for both biofilms C and REF. 

It is noteworthy that after the contact of pumpkin with the packaging, in A and B samples at 11 days, new resonances appeared (doublet at 1.15 ppm) due to compounds not present in the fresh pumpkin. Selective TOCSY experiment indicated its correlations with 3.44, 3.55, and 3.89 ppm resonances, and all of them were assigned to propylene glycol. In a control experiment, packaging A was put in contact with deuterated water followed by acquisition of ^1^H NMR spectrum. The presence of all assigned signals of propylene glycol was confirmed; moreover, even more intense signals of glycerol were observed at 3.56, 3.66 and 3.79 ppm. Therefore, propylene glycol and glycerol from A and B packaging migrated in the pumpkin samples during storage. However, these compounds are allowed in foods and their presence in the food packaging depend on the industrial process applied for biofilm production. 

#### 3.2.2. Total Carotenoids, Chlorophylls a and b, Total Polyphenols and Antioxidant Activity

**Figure 5:**
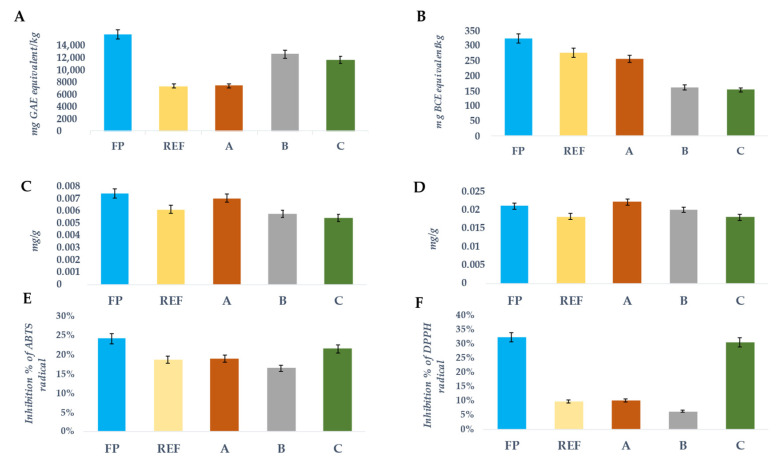
Histograms of total phenolic content (**A**) expressed in mg GAE/kg ± SD; total carotenoid content (**B**) expressed in mg BCE/kg± SD; chlorophyll a (**C**) and chlorophylls b (**D**) content expressed in mg/g± SD; ABTS (**E**) and DPPH (**F**) assays expressed as Inhibition %± SD, evaluated in fresh and packaged pumpkin samples, during shelf-life. FP: Fresh pumpkin (light blue trace); REF: polyethylene (yellow trace); A: biodegradable cellophane from regenerated cellulose (orange trace); B: biofilm obtained from maize starch, cassava, eucalyptus (grey trace); C: poly-lactate obtained from maize starch (green trace).

The pigments content (total carotenoids, chlorophylls a and b), total polyphenols and antioxidant activity was analysed in both fresh and packaged pumpkin samples at time = (t_0_) and 11 days (t_11_) and the results are displayed in Figure 5. In general, it is possible to note a decrease in all these bioactive compounds in the packaged products compared to the fresh one.

Regarding the chlorophyll content in pumpkin samples stored in different biofilms the results showed that the chlorophyll b concentration exceeded that of chlorophyll a by an average of 68% (the values ranged from 64% for the fresh samples (t_0_) to 71% for packaging B (t_11_)) (*p* < 0.01). By comparing the different biofilms with respect to the REF, the highest level of chlorophyll a and chlorophyll b was detected in the packaging A sample, even if this difference did not reach statistical significance (Figure 5C,D).

A similar variability was also evaluated for the total polyphenols, TPC (Figure 5A) and for the total carotenoids, TCC (Figure 5B). During the 11-day storage period, REF and A—packaged pumpkin samples showed a reduction about 56% and 53%, respectively (REF: 6141.73 ± 0.06 mg GAE/kg, and A: 6210.57 ± 0.03 mg GAE/kg), compared with the fresh samples at t_0_ (13,255.25 ± 0.02 mg GAE/kg). Regarding the total amount of carotenoids in pumpkin samples during shelf-life, the results showed that TCC decreased more than 53%, especially in B and C packaged pumpkin samples (160.12 ± 1.36 mg BCE/kg, and 152.98 ± 1.1 mg BCE/kg); while, in REF packaged pumpkin, the content of carotenoids was almost unaltered towards fresh pumpkin (276.24 ± 2.27 mg BCE/kg). The antioxidant activity was measured by two widely used methods, DPPH and ABTS which are based on free radical scavenging and reducing power abilities of different type of antioxidant compounds found in plant food extracts. ABTS radical scavenging potential was comparatively higher than the DPPH radical scavenging activity, especially in polyethylene, A- and B-packaged pumpkin samples. ABTS is mainly oxidized by peroxyl radicals and it is soluble in both aqueous and organic solvents, so can be used to determine both hydrophilic and lipophilic antioxidant capacity (AOC) of extracts. While DPPH is a stable nitrogen radical that bears no resemblance to peroxyl radicals involved in lipid peroxidation. The decolourisation of the reagent occurs by both radical reaction and reduction, and steric accessibility is a determining factor in the reaction. Therefore, small molecules that have better access to the radical site have a higher AOC with this test [35]. Consequently, the combined use of these two assays provided an effective evaluation of antioxidant activity. The pumpkin samples stored in packaging C at t_11_ seemed to better preserve antioxidant activities both for ABTS and DPPH assays (21.47% and 30.34%, respectively) compared to fresh ones (24.02% and 32.10%) (Figure 5E,F). Different authors observed that the content of bioactive compounds in plant-origin foods, such as pumpkin, strictly de pended on storage period, plant varieties and post-harvest conditions [36,37].

#### 3.2.3. Biogenic Amines Content 

**Figure 6:**
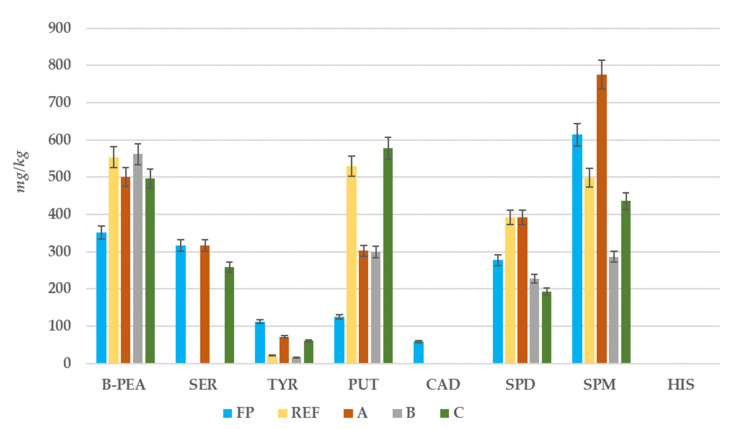
Biogenic amines content in fresh (t_0_) and packaged pumpkin samples (t_11_). FP: Fresh pumpkin (light blue trace); REF: polyethylene (yellow trace); A: biodegradable cellophane from regenerated cellulose (orange trace); B: biofilm obtained from maize starch, cassava, eucalyptus (grey trace); C: poly-lactate obtained from maize starch (green trace). β-PEA: β-phenylethylamine; PUT: putrescine; CAD: cadaverine; HIS: histamine; SER: serotonin; TYR: tyramine; SPD: spermidine; SPM: spermine.

The content of eight BAs was evaluated in fresh and packaged pumpkin samples by high-performance liquid chromatography with fluorescence detection. The BAs investigated were spermine (SPM), spermidine (SPD), putrescine (PUT), and cadaverine (CAD) for polyamines, whereas β-phenylethylamine (β-PEA), histamine (HIS), serotonin (SER), and tyramine (TYR) were studied for monoamines. Figure 6 exhibited different trends of BA content for fresh and packaged pumpkin samples during the 11-day storage. 

All BAs investigated were found in pumpkin samples in a great variability, except for HIS, an amine with negative effects on health, which was not detected in any samples. Besides, TYR, another dangerous BA for human health was detected in all samples, and the fresh pumpkin presented the highest amount (112.24 ± 0.01 mg/kg). In plant-origin foods, tyramine content might be associated with microbial aminogenic activity [38]. While, during the storage period, TYR content was reduced in all type of packaged samples, especially in the packaging REF and B (20.68 ± 0.52 mg/kg, and 15.92 ± 0.90 mg/kg). Different authors observed that refrigeration temperatures (4–10 °C), delays or reduces the aminogenic potential of microorganisms, which could have been responsible for the formation of monoamines, such as TYR and HIS, during storage period [39,40].

### 3.3. Microbiological Analysis 

Below are the results of the microbiological analysis together with mass loss, water activity, pH value, and carbon dioxide headspace concentration evaluation.

#### 3.3.1. Mass Loss 

Both moisture content and water activity are important in formulating products for safety and stability. Moisture content of fresh pumpkin was determined by weight loss upon drying equal to 95.58 ± 0.14% wet basis.

**Figure 7:**
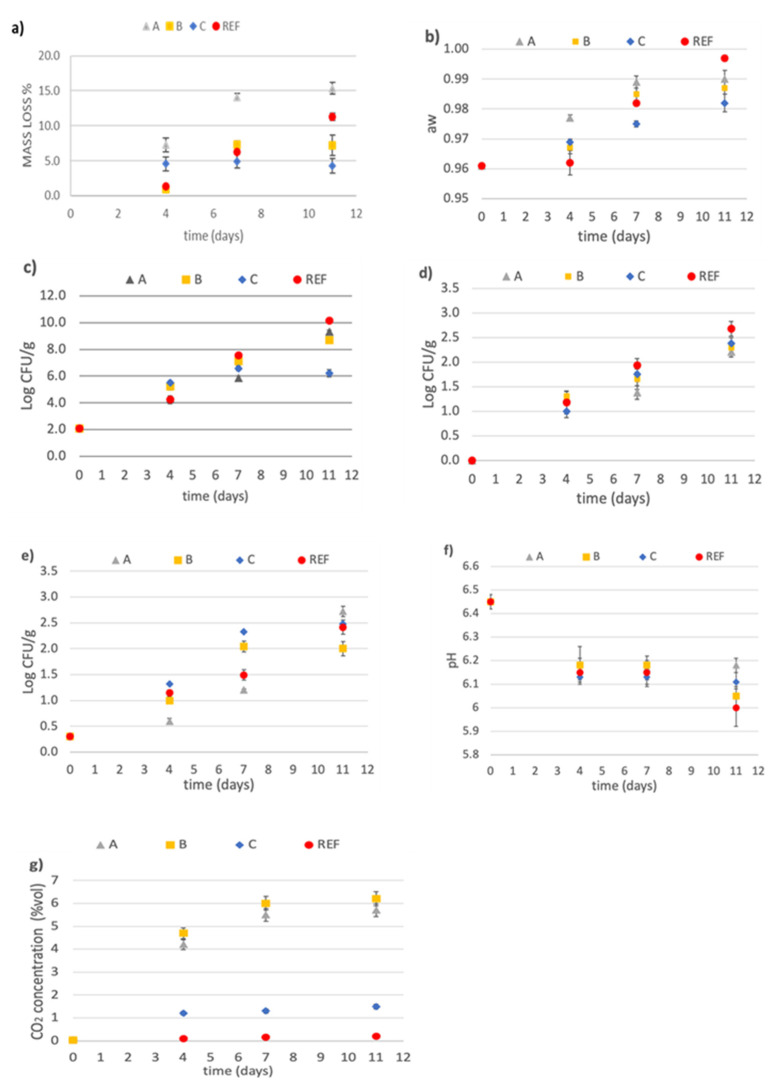
(**a**) Mass loss percentage, (**b**) water activity (a_w_), (**c**) total microbial count, (**d**) lactic acid bacteria load, (**e**) yeasts and molds count, and (**f**) pH evaluated in fresh and packaged pumpkin samples, during shelf-life; (**g**) carbon dioxide concentration in headspace of pumpkin package in different films. REF: Polyethylene film; commercial biofilms: A, B and C.

In Figure 7a, the pumpkin mass loss was plotted as a function of storage time for all the films investigated. The lower weight loss occurred for the biofilm C: its value remained constant at 4.58 ± 0.32% up to 11 days of storage at 5 °C. After 11 days, similar values were observed for biofilm B (7.24 ± 0.05%), and lower than those obtained for the polyethylene film. Only for biofilm A the weight loss was higher (15.36 ± 0.87%) than REF film.

#### 3.3.2. Water Activity Evaluation 

Water activity, a_W_, is a measure of how much of that water is free, i.e., unbound, and thus available to microorganisms to use for growth. Therefore, it is important with regard to food safety. Microorganisms will not grow below a certain water activity level—a_W:_ 0.90 for most pathogenic bacteria, 0.70 for spoilage molds, and 0.60 for all microorganisms. While temperature, pH, oxygen availability, and several other factors can influence whether an organism will grow in a product and at what rate, water activity is often the most important factor. 

**Figure 8:**
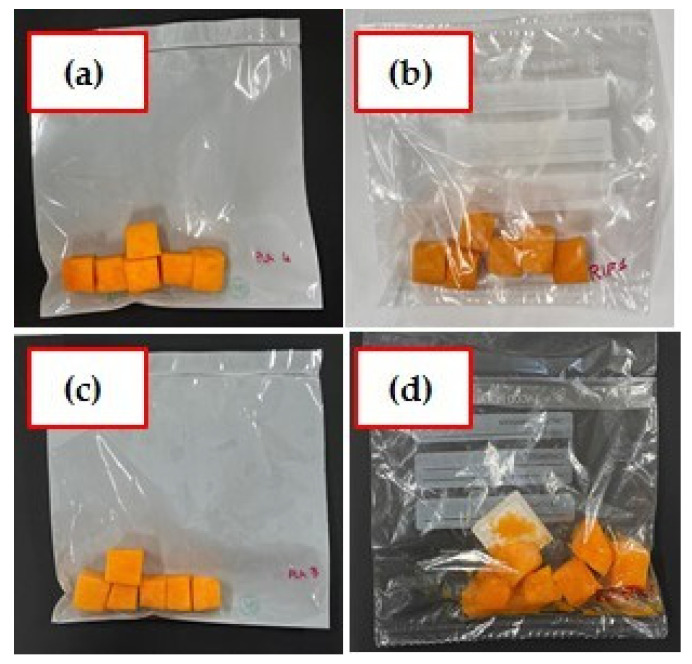
Pictures of pumpkins stored in biofilm C and in REF packaging at time 0 and after 11 days. (**a**) C at 0 days; (**b**) REF at 0 days; (**c**) C at 11 days; (**d**) REF at 11 days.

The water activity of fresh pumpkin was of 0.961 ± 0.001 and increased during the storage time for all the packages, especially for polyethylene film (Figure 8b), with values typical of high moisture foods in the range of 0.900 to 0.999 [41]. These conditions promoted the bacterial growth. At 11 days in the samples conserved in polyethylene film, the presence of water on the surface of the pumpkin cubes was observed (Figure 8). This caused deterioration and loss of product quality. On the contrary, the a_w_ measured in biofilm C reached a value of 0.982 ± 0.003, slightly lower than the other biofilms. 

#### 3.3.3. Microbiological Analysis

The total microbial count was plotted as a function of storage time in Figure 7c for all the tested packaging systems. The total count was below the threshold value (8.00 Log CFU/g) [42,43] up to 7 days of storage for all the films tested, and 11 days only for biofilm C, suggesting that microbial growth did not limit the shelf life of the pumpkin in this time interval. The analysis of variance showed significant differences between samples at different times. In comparison, fresh cut pumpkins last for 2/3 days in refrigerators. 

The lactic acid bacteria, and yeasts and molds counts were plotted as the function of storage time and reported in Figure 7d,e, respectively. In all samples, the values of microbial loads were always under the acceptability limit of 4.00 Log UFC/g [42] during the 11 days. The lactic acid bacteria load increased during the time up to 11 days; the values obtained with the different biofilms were similar, but lower than those obtained for polyethylene. It was probably the pH conditions at 4 days of storage (Figure 7f) that promoted the growth of lactic acid bacteria, which tolerate acidic conditions and are endogenous microbiota of the pumpkin [44].

For the yeasts and molds load (Figure 7e), an increasing value was observed in the first week which was maintained constant in the biofilm B up to 11 days (2.00 log CFU/g), but continuously increased in the other films. This behavior can be ascribed to the barrier effect of the biofilm B, higher than other films, and the consequent lower oxygen concentration in the package atmosphere, which avoids the growth of the aerobic organisms.

#### 3.3.4. pH Value

pH values of pumpkin packaged in the four different films are shown in Figure 7f. The pH value of fresh pumpkin was 6.45 ± 0.03. In the first 4 days, a pH decreasing trend was observed for all the samples. The variations of pH among samples were not statistically significant up to 7 days. After 7 days, for biofilms C and A, the pH remained almost constant, 6.11 ± 0.02 and 6.18 ± 0.02, respectively. 

The observed decreasing trend in the pH during the storage is a possible consequence of the accumulation of lactic acid produced in the lactic acid bacteria metabolism [45]. Moreover, the observed increase in CO_2_ concentrations (Figure 7g) measured in the headspace of package can also contribute to a decrease in pH [46].

#### 3.3.5. Carbon Dioxide Headspace Concentration 

The headspace carbon dioxide concentration during shelf life of the packaged pumpkins is reported in Figure 7g. For both biofilms A and B, an increase was observed in carbon dioxide concentration from 0.04% to 5.70% vol in the first 4 days, and then it remained constant up to 11 days. This increase was higher than that measured for biofilm C and polyethylene film, and in agreement with the measured CO_2_ transmission rate obtained from the permeation tests.

#### 3.3.6. Aroma Compounds

The aroma compounds of the pumpkin stored in biofilm C at the initial state (t = 0) and after 11 days were determined by headspace extraction followed by GC–MS analysis. The compounds were identified by comparison of mass spectra with NIST libraries. The elution order of the compounds agreed with the literature data [47].

Nineteen aroma compounds, including alcohols, esters, aldehydes and alkenes, were identified and reported in Table 5 with their retention times. The peak areas of the identified compounds were calculated by integration for both samples, and the difference in percentage between t = 0 and 11 days was valuated (ΔArea, %). After 11 days of storage in biofilm C, a general decreasing trend was observed in the aroma compounds with a maximum loss in hexanal.

### 3.4. T_1_ and T_2_ Relaxation Times of Fresh Pumpkin Packaged in Biofilm B and C by Portable TD-NMR

After 1 day in both packaging, the longitudinal magnetization decay of fresh packaged pumpkin showed a mono-exponential trend with a constant time of about 1.5–2 s. On the contrary, the transverse decay of the magnetization showed a bi-exponential trend due to the presence of water in two different proton domains. A long component of T_2_ was found to be of about 35–40 ms affecting most of the spin protons (between 90–92% of the protons, depending on the sample), and a short component of T_2_ of about 9–11 ms affecting the 8–10% of the protons. In food and plants cells, water molecules can experience particular physical and chemical environments due to various cell compartments and organelles, and by interacting with different macromolecules [48,49,50,51]. Consequently, the decay of magnetization is usually described by a multi-exponential function in which T_1_ and T_2_ show more than one component. However, water molecules or protons are in exchange between these compartments, and T_1_ and T_2_ relaxation times can be averaged by depending on both the exchange and the relaxation rates. In such a dynamic system, the direct assignment of relaxation times to particular compartments is a difficult task to be reached. 

In fresh pumpkin, a single averaged T_1_ value was found, indicating an exchange among water molecules from different compartments of pumpkin tissue. T_2_ measurements yield a more detailed information on the partitioning of water in vegetable tissue than T_1_. According to the literature, the two values of T_2_ found in pumpkin may be categorized into “free water’’ mostly due to protons that shall be free-moving into vacuole, and “hindered water” possible due to water molecules that exchange across the other compartments such as cytoplasm, cell water/extracellular water. 

**Figure 9:**
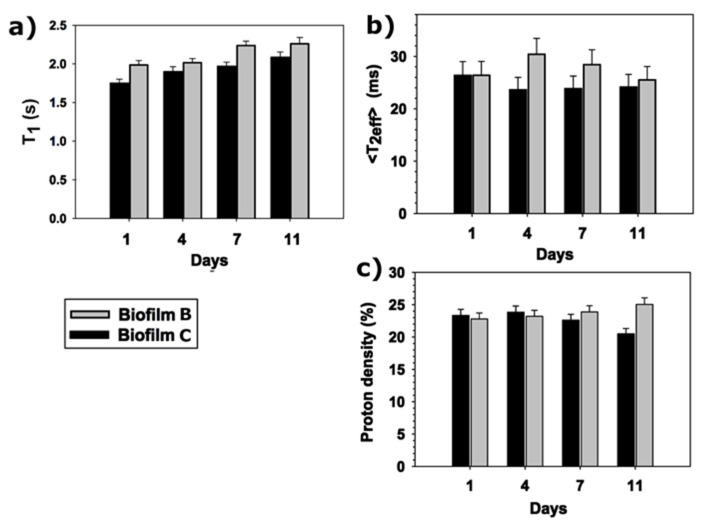
(**a**) Longitudinal relaxation times of packaged pumpkin; (**b**) mean transverse relaxation times and (**c**) proton density of packaged pumpkin in biofilm B and biofilm C as function of storage time.

During the storage period, both T_1_ and T_2_ were collected, see Figure 9. Because of the high variability of T_2_ values collected during the storage time, and between each single pumpkin sample, the arithmetic mean of the relaxation times <T_2_> was calculated and compared to the T_1_ values, and to the total proton density. The T_1_ showed a slight increase during the storage period both in biofilm B and C. On the contrary, depending on the biofilm type, a different trend can be observed in the T_2_. Biofilm B showed an initial elongation of the T_2_, indicating a greater water mobility, followed by a decrease of T_2_ value. In biofilm C, the T_2_ values are rather stable expect to an initial shortening, indicating a slight decrease in the water mobility. Biofilm B and C showed a different behavior also in the proton density. For 7 days in both biofilms, the proton density was found to be quite constant. After the first week, the pumpkin in biofilm C showed a decrease in the proton density, whereas it showed a slow increase in biofilm B, possible indicating a higher water content at the sample surface.

These preliminary results indicate that biofilm B is less effective in preserving the mobility of water in fresh pumpkin compared to the biofilm C. However, many processes may affect the relaxation processes of water molecules in pumpkin during the storage, including the possible changes in cell membrane permeability, the diffusion of O_2_ and CO_2_ in the pumpkin tissue, and the variation in water activity and in food microstructure. Further investigations will be needed to correlate T_1_, and T_2_ values to other parameters used to assess the effectiveness of food packaging. 

## 4. Conclusions

A comparison between different commercial biofilms for the preservation of pumpkin was reported. Among the films investigated, B film, based on Corn starch, cassava and eucalyptus, showed more ductile and deformable behavior, indicating better mechanical flexibility requirements for packaging applications, which is crucial to avoid breaking during processing and use. Moreover, biofilm B and A provided proper requirements in terms of oxygen and carbon dioxide transmission rate for food packaging applications.

Biofilm functionality was investigated through a multimethodological protocol consisting of chemical and microbiological analyses applied to fresh and packaged samples at different times.

Untargeted NMR spectroscopy allowed to obtain a comprehensive metabolite profiling of packaged and unpackaged pumpkin samples: organic acids, ammino acids, and sugars were identified and quantified showing a sucrose increase and a malic acid decrease in all the examined biofilms respect to FP; the other major sugars (fructose, glucose, galactose) levels remained quite steady in biofilms B and C; the most abundant amino acids (aspartate, glutamine, asparagine, arginine, pyro-glutamate) remained quite constant in biofilm A and decreased significantly in biofilm B.

Targeted methodologies allowed polyphenols, anthocyanins, carotenoids, volatile compounds, fatty acids and potential contaminants (i.e., biogenic amines as histamine, tyramine, etc.) to be tested and monitored. 

In general, it is possible to note a trend of pigments decreasing in the packaged products compared to the fresh ones. After 11 days the content of total carotenoids tends to remain constant in the REF film compared to the fresh product, while it tends to decrease in biofilms A, B and especially in C.

After an 11-day period, A and C packaged samples showed the highest amount of total biogenic amines, especially for spermine, spermidine and putrescine, which could be considered as an index of food spoilage. Whereas the trends of TPC, antioxidant activities and TCC all decreased during the storage period, resulting in a significant loss of 56% for total phenolic content and 53% of total carotenoids content, especially in the REF and A packaged pumpkin samples. Regarding the chlorophylls content, the highest level of chlorophyll *a* and chlorophyll *b* was detected in the packaging A sample compared to the REF one.

Water activity was also measured to have indication of unbound water, which is thus available to microorganisms to use for growth. It is thus important with regard to food safety. The water activity of fresh pumpkin was of 0.961 ± 0.001 and increased during the storage time for all the packages, especially for polyethylene film, with water activity levels at which all microorganisms may grow. 

Microbiological analyses were also carried out to assess the product quality. Microbiological analysis results suggest that microbial growth did not limit the shelf-life of the pumpkin up to 7 days for the biofilms A and B and up to 11 days for the biofilm C. The lactic acid bacteria and yeasts and molds counts increased but were for all packages below the acceptability limit during the 11 days. In particular, for biofilm B the yeasts and molds load increased in the first week and then was maintained constant up to 11 days. These are very promising results considering that fresh cut pumpkins last for 2/3 days in refrigerators.

The observed decreasing trend in the pH during the storage in all packages is a possible consequence of the accumulation of lactic acid produced in the lactic acid bacteria metabolism. Meanwhile, the lower increase of yeast and molds in biofilm B can be due to the observed increase in CO_2_ concentrations measured in the headspace of package. In detail, for both biofilms A and B it was observed an increase in carbon dioxide concentration from 0.04% to 5.70% vol in the first 4 days, and then it remained constant up to 11 days. This increase is higher than that measured for biofilm C and polyethylene film. It is argued that the barrier effect of the biofilm B, higher than other films, and the consequent lower oxygen concentration in the package atmosphere, avoids the growth of the aerobic organisms.

In the case of samples packed with C film that turned to be the most promising packaging for microbiological point of view, the aroma analysis was also carried out by headspace extraction followed by GC–MS analysis. Nineteen compounds, including alcohols, esters, aldehydes and alkenes, were identified showing a general, but not too drastic, decreasing trend after 11 days.

The water dynamics were studied by time-domain NMR portable relaxometry. T_1_ and T_2_ relaxation times were measured in fresh and packaged samples pumpkin. A single averaged T_1_ value was found in fresh and packaged pumpkin indicating an exchange among water molecules from different compartments of pumpkin tissue. During the whole period of preservation (11 days), T_1_ showed a similar trend in both biofilm B and C with an increase of T_1_ values during the storage period. In contrast, two values of T_2_ were found in pumpkin possibly attributable to “free water” (water molecules free moving into vacuole), and “hindered water” (water molecules exchanging across the other compartments). The observed T_2_ behavior in packaged sample during 11 days of conservation was depending on the biofilm type. Biofilm B showed an initial elongation of the T_2_, indicating a greater water mobility, followed by a decrease of T_2_ value. In biofilm C, the T_2_ values show a stable trend after an initial shortening, indicating a slight decrease in the water mobility. In terms of water dynamics these preliminary results indicate that biofilm B is less effective in preserving the mobility of water in fresh pumpkin compared to the biofilm C. 

Overall, results show that the biofilm C is the more suitable for applications of pumpkin in terms of the preservation of food quality properties (i.e., sugar content, organic acids, aminoacids, fatty acids in the organic fraction and aroma compounds). In contrast to A and B biofilms, pumpkin samples within C package resonances of propylene glycol (not present in the fresh pumpkin) were not observed.

Moreover, during the storage it was observed that in all the examined biofilms the total phenolic content and total carotenoids of pumpkin decreased compared to FP, but the biofilm C showed a better preservation of antioxidant activity.

Biofilm C also assured a low level of microorganisms’ growth up to 11 days and a low amount of total biogenic amines which are an index of food spoilage. 

In terms of water dynamics, the results of portable TD- NMR indicate that biofilm B is less effective in preserving the mobility of water in fresh pumpkin compared to the C biofilm.

Regarding mechanical properties, biofilm C has a good mechanical strength, but lower mechanical flexibility.

## Figures and Tables

**Table 1:** Composition of commercial biofilms, as reported on the package label, and measured thickness, oxygen transmission rate (OTR) and carbon dioxide transmission rate (CO_2_TR).

Biofilm Commercial	Biofilm Composition	Thickness (µm) (Measured Value)	OTR (cm^3^⸱m^−2^⸱day^−1^) (Measured Value)	CO_2_TR (cm^3^⸱m^−2^⸱day^−1^) (Measured Value)
A	Biodegradable cellophane from regenerated cellulose pulp	20	4.91 ± 0.27	10.78 ± 0.50
B	Corn starch, cassava and eucalyptus	40	4.30 ± 0.20	13.42 ± 0.71
C	Polylactic acid from corn starch	30	646.41 ± 32.33	2162.51 ± 110.13

**Table 2:** NMR signals selected for quantitative NMR analysis (qNMR).

ppm	Compound	ppm	Compound
0.94	Isoleucine	4.18	Pyro-glutamate
0.97	Leucine	4.30	Malic acid
1.05	Valine	4.60	β-Galactose
1.34	Threonine	4.66	β-Glucose
1.49	Alanine	5.25	α-Glucose
1.70	Arginine	5.28	α-Galactose
2.30	GABA	5.42	Sucrose
2.35	Glutamic acid	6.53	Fumaric acid
2.46	Glutamine	6.91	Tyrosine
2.54	Citric acid	7.44	Phenylalanine
2.81	Aspartic acid	7.55	Tryptophan
2.89	Asparagine	8.15	Istidine
3.21	Choline	8.47	Formic acid
3.30	Myo-inositol	8.84	Trigonelline
4.12	Fructose		

**Table 3:** Carbohydrate, organic acid, amino acid and other metabolite amounts (mg/10 g ± SD) by NMR analysis of the pumpkins Bligh–Dyer hydroalcoholic fractions reported for fresh and packaged pumpkin samples. FP = fresh pumpkin sample (time 0); A, B and C = commercial biofilms; REF = polyethylene film. Analyses were carried out in duplicate.

	FP	A	B	C	REF
**Carbohydrates**					
Sucrose	759.42 ± 21.09	1425.34 ± 44.62	1026.61 ± 20.09	989.41 ± 26.60	1113.97 ± 87.08
Fructose	1095.69 ± 34.88	823.90 ± 26.82	1116.69 ± 36.09	1005.36 ± 9.47	1169.32 ± 135.70
Glucose	987.47 ± 36.58	641.06 ± 31.04	1053.60 ± 9.43	879.11 ± 15.30	1025.42 ± 125.89
Mio-inositol	170.66 ± 9.00	165.97 ± 2.98	147.79 ± 6.82	156.90 ± 5.16	162.15 ± 15.43
Galactose	80.97 ± 6.46	53.74 ± 2.68	67.18 ± 1.46	63.35 ± 0.58	70.46 ± 7.06
**Organic acids**					
Malic acid	340.84 ± 1.41	301.09 ± 5.97	255.01 ± 2.20	277.07 ± 1.38	178.78 ± 1.05
Citric acid	20.52 ± 0.77	34.76 ± 0.72	18.82 ± 0.24	36.14 ± 4.27	22.54 ± 0.11
Fumaric acid	0.94 ± 0.09	1.10 ± 0.04	1.81 ± 0.14	0.80 ± 0.05	1.35 ± 0.06
Formic acid	0.59 ± 0.19	0.69 ± 0.12	0.89 ± 0.06	0.73 ± 0.06	0.78 ± 0.10
**Amino acids**					
Aspartate	491.06 ± 16.94	456.09 ± 5.84	322.02 ± 2.76	457.88 ± 19.53	532.23 ± 47.67
Glutamine	272.77 ± 15.89	259.64 ± 4.08	196.37 ± 6.30	240.50 ± 5.44	286.30 ± 32.78
Asparagine	297.37 ± 8.98	284.24 ± 5.11	256.66 ± 2.86	238.47 ± 0.27	249.83 ± 20.12
Arginine	71.17 ± 0.20	70.62 ± 1.36	51.75 ± 0.78	63.40 ± 1.15	44.85 ± 4.30
Pyro-glutamate	57.24 ± 5.45	51.39 ± 2.01	37.13 ± 0.87	58.49 ± 8.72	40.31 ± 3.40
Tyrosine	55.17 ± 1.02	48.70 ± 0.70	35.85 ± 0.24	48.02 ± 0.45	46.05 ± 4.60
Alanine	12.47 ± 0.58	8.79 ± 0.07	43.42 ± 2.31	35.32 ± 1.35	9.47 ± 0.92
Glutamate	19.83 ± 1.58	15.46 ± 0.08	18.37 ± 1.80	36.07 ± 3.02	17.52 ± 1.33
GABA	29.99 ± 1.38	30.80 ± 1.94	28.03 ± 0.04	26.34 ± 0.05	24.13 ± 2.54
Phenylalanine	21.90 ± 0.63	20.26 ± 0.32	15.88 ± 0.17	20.90 ± 0.21	20.65 ± 1.82
Isoleucine	16.00 ± 1.04	18.07 ± 0.50	14.87 ± 0.59	12.71 ± 0.13	12.78 ± 1.71
Histidine	14.44 ± 0.13	15.99 ± 0.04	11.64 ± 0.76	13.07 ± 0.07	11.64 ± 1.40
Valine	16.22 ± 0.90	16.77 ± 0.46	13.82 ± 0.15	13.52 ± 0.24	13.54 ± 1.59
Threonine	15.87 ± 0.78	15.81 ± 0.22	14.83 ± 0.65	14.18 ± 0.01	15.18 ± 1.57
Tryptophan	12.31 ± 0.20	12.69 ± 0.75	8.50 ± 0.04	12.94 ± 0.10	10.33 ± 1.14
Leucine	2.04 ± 0.14	2.35 ± 0.11	2.02 ± 0.05	1.54 ± 0.05	1.12 ± 0.24
**Miscellaneous metabolites**					
Trigonelline	10.99 ± 0.30	9.49 ± 0.46	9.01 ± 0.43	14.65 ± 0.18	8.39 ± 0.68
Choline	9.99 ± 0.49	7.98 ± 0.14	6.23 ± 0.02	9.50 ± 0.24	7.66 ± 0.75

**Table 4:** Fatty acid percentages (molar % ± SD) by NMR analysis of the pumpkins Bligh–Dyer organic fractions reported for fresh and packaged pumpkin samples. FP = fresh pumpkin sample (time 0); A, B and C = commercial biofilms; REF = polyethylene film. Analyses were carried out in duplicate.

	FP	A	B	C	REF
Tot SFA	63.47 ± 0.92	58.90 ± 1.84	62.56 ± 0.78	62.69 ± 0.79	63.97 ± 3.51
Tot UFA	36.53 ± 0.92	41.10 ± 1.84	37.44 ± 0.78	37.31 ± 0.79	36.04 ± 3.51
TUFA	23.31 ± 0.17	25.52 ± 0.37	25.44 ± 0.25	25.23 ± 0.17	27.81 ± 1.26
MUFA	5.19 ± 1.13	7.19 ± 2.60	3.45 ± 1.10	4.88 ± 0.56	2.79 ± 2.20
DUFA	8.02 ± 0.04	8.40 ± 0.39	8.56 ± 0.07	7.20 ± 0.05	5.43 ± 0.05

**Table 5:** Volatile flavor compounds identified in pumpkin samples in C-PLA film and percentage variation of peak area recorded at 11 days at 5 °C.

Compound	Retention Time (min)	Δ Area, %
Ethanol	2.560	22.23 ± 0.12
Hexanal	3.951	−31.50 ± 1.23
Ethyl acetate	4.998	1.05 ± 0.24
1-Hexanol	5.347	−15.03 ± 2.87
1-Octen-3-ol	8.162	−14.00 ± 2.26
3-Hexen-1-ol	9.032	−11.71 ± 1.58
2 cyclohexen-1-ol-2.4.4-trimethyl	10.223	−5.10 ± 1.47
2-octenal	10.388	14.47 ± 1.51
iso-phorone	10.445	8.90 ± 1.04
Eucalyptol	10.765	−12.36 ± 1.78
6-nonenal	11.681	17.40 ± 1.24
2–6 nonadienal	13.123	−22.47 ± 0.60
6-nonen-1-ol	13.621	−27.11 ± 0.41
Decanal	14.616	13.49 ± 0.17
1-cyclohexene-1-carboxaldehyde-2,6,6-trimethyl	15.068	−6.32 ± 0.04
1-cyclohexene-1-acetaldehyde-2,6,6-trimethyl	16.081	−7.45 ± 0.02
Tetradecane	19.806	6.64 ± 0.05
Naftalene 1,4-dimethyl	20.281	−0.50 ± 0.06
3-buten-2-one,4-(2,6,6-trimethyl-1-cyclohexen-1-yl)	20.544	−26.14 ± 0.01
5,9-undecadien-2-one,6,10-dimethyl	21.071	−11.47 ± 0.09

## Data Availability

The data presented in this study are available on request from the corresponding authors.

## Supplementary Materials

The following are available online at https://www.mdpi.com/article/10.3390/foods10102440/s1, Figure S1: Bar charts of the amino acids identified and quantified (mg/10 g of DW ± SD) in the 1H NMR spectra of hydroalcoholic extracts of freeze-dried pumpkins samples. FP = Fresh pumpkin sample (time 0); A, B and C = commercial biofilms; REF = polyethylene film. Figure S2: Bar charts of the sugars (a), organic acids (b) and other metabolites (c) identified and quantified (mg/10 g of DW ± SD) in the 1H NMR spectra of hydroalcoholic extracts of pumpkins. FP = Fresh Pumpkin sample (time 0); A, B and C = commercial biofilms; REF= polyethylene film. Figure S3: Bar charts of the fatty acids identified and quantified (molar % ± SD) in the 1H NMR spectra of organic extracts of pumpkins. FP = Fresh Pumpkin sample (time 0); A, B and C = commercial biofilms; REF= polyethylene film. Table S1: Metabolites identified in the 600.13 MHz 1H NMR, 1H-1H TOCSY, 1H-13C HSQC, and 1H-13C HMBC spectra of Bligh–Dyer hydroalcoholic extracts of pumpkins in phosphate buffer/D2O acquired at 28 °C. Asterisks (*) indicate signals selected for integration. Table S2: Metabolites identified in the 600.13 MHz 1H NMR, 1H- 1H TOCSY, 1H-13C HSQC, and 1H-13C HMBC spectra of Bligh–Dyer organic extracts of pumpkins CDCl3/CD3OD (2:1 *v*/*v*) mixture acquired at 28 °C. Asterisks (*) indicate signals selected for integration. For the integration of total fatty acids (IFA) and total unsaturated fatty acids (IUFA), the region of 2.20–2.36 ppm and 5.25–5.40 ppm, respectively, were considered. Table S3: One-way analysis of variance (ANOVA) of the resulted NMR quantification of the polar metabolites. F and *p* values for the different examined packaging that reach a *p* < 0.05 respect to the FP were showed. Table S4: One-way analysis of variance (ANOVA) of the resulted NMR quantification of the fatty acids. F and *p* values for the different examined packaging that reach a *p* < 0.05 respect to the FP were showed.

## References

[1] Lima K.S., Sanches A.G., Cordeiro C.A.M. (2019). Evalution of packaging in the conservation of minimally processed squash. Appl. Res. Agrotechnol. Guarapuava-PR.

[2] Habibunnisa R., Baskaran R., Prasad K., Mysore S. (2001). Storage behaviour of minimally processed pumpkin (Cucurbita maxima) under modified atmosphere packaging conditions. Eur. Food Res. Technol..

[3] Lucera A., Simsek F., Conte A., Del Nobile M.A. (2012). Minimally processed butternut squash shelf life. J. Food Eng..

[4] Ingallina C., Maccelli A., Spano M., Di Matteo G., Di Sotto A., Giusti A.M., Vinci G., Di Giacomo S., Rapa M., Ciano S. (2020). Chemico-Biological Characterization of Torpedino Di Fondi^®^ Tomato Fruits: A Comparison with San Marzano Cultivar at Two Ripeness Stages. Antioxidants.

[5] Mannina L., Sobolev A.P., Viel S. (2012). Liquid state 1H high field NMR in food analysis. Prog. Nucl. Magn. Reson. Spectrosc..

[6] ISO 527-3:2008 Plastics—Determination of Tensile Properties Test Conditions for Films and Sheets. https://www.iso.org/standard/70307.html.

[7] ISO 15105-2:2003 Plastics—Film and Sheeting—Determination of Gas-Transmission Rate—Part 2: Equal-Pressure Method. https://www.iso.org/standard/37514.html.

[8] Ingallina C., Sobolev A.P., Circi S., Spano M., Fraschetti C., Filippi A., Di Sotto A., Di Giacomo S., Mazzoccanti G., Gasparrini F. (2020). *Cannabis sativa* L. inflorescences from monoecious cultivars grown in central Italy: An untargeted chemical characterization from early flowering to ripening. Molecules.

[9] Sobolev A.P., Segre A., Lamanna R. (2003). Proton high-field NMR study of tomato juice. Magn. Reson. Chem..

[10] Sobolev A.P., Mannina L., Capitani D., Sanzò G., Ingallina C., Botta B., Fornarini S., Crestoni M.E., Chiavarino B., Carradori S. (2018). A multi-methodological approach in the study of Italian PDO “Cornetto di Pontecorvo” red sweet pepper. Food Chem..

[11] Lichtenthaler H.K., Buschmann C. (2001). Chlorophylls and Carotenoids: Measurement and Characterization by UV-VIS Spectroscopy. Curr. Protoc. Food Anal. Chem..

[12] Wellburn A.R. (1994). The Spectral Determination of Chlorophylls a and b, as well as Total Carotenoids, Using Various Solvents with Spectrophotometers of Different Resolution. J. Plant Physiol..

[13] Rapa M., Ciano S., Gobbi L., Ruggieri R., Vinci G. (2021). Quality and safety evaluation of new tomato cultivars. Ital. J. Food Sci..

[14] Rapa M., Ciano S., Ruggieri R., Vinci G. (2021). Bioactive compounds in cherry tomatoes (*Solanum lycopersicum* var. Cerasiforme): Cultivation techniques classification by multivariate analysis. Food Chem..

[15] Preti R., Rapa M., Vinci G. (2017). Effect of streaming and boiling on the antioxidant properties and biogenic amines content in green bean (*Phaselous vulgaris*) varieties of different colours. J. Food Qual..

[16] Liu F.X., Fu S.F., Bi X.F., Chen F., Liao X.J., Hu X.S., Wu J.H. (2013). Physico-chemical and antioxidant properties of four mango (*Mangifera indica* L.) species in China. Food Chem..

[17] Blümich B., Perlo J., Casanova F. (2008). Mobile single-sided NMR. Progr. Nucl. Magn. Reson. Spectrosc..

[18] Farrar T.C., Becker E.D. (1971). Pulse and Fourier Transform NMR.

[19] Stark N.M. (2016). Opportunities for cellulose nanomaterials in packaging films: A review and future trends. J. Renew. Mater..

[20] Siracusa V., Blanco I., Romani S., Tylewicz U., Rocculi P., Rosa M.D. (2012). Poly (lactic acid)-modified films for food packaging application: Physical, mechanical, and barrier behavior. J. Appl. Polym. Sci..

[21] Arrieta M.P., López J., Ferrándiz S., Peltzer M.A. (2013). Characterization of PLA-limonene blends for food packaging applications. Polym. Test..

[22] Embleni A. (2013). Modified atmosphere packaging and other active packaging systems for food, beverages and other fast-moving consumer goods. Trends in Packaging of Food, Beverages and Other Fast-Moving Consumer Goods (FMCG).

[23] Siracusa V. (2012). Food packaging permeability behaviour: A report. Int. J. Polym. Sci..

[24] Chinga-Carrasco G., Syverud K. (2012). On the structure and oxygen transmission rate of biodegradable cellulose nanobarriers. Nanoscale Res. Lett..

[25] http://www.ecosign-project.eu/wp-content/uploads/2018/09/FOOD_UNIT11_IT_Lecture.pdf.

[26] Piscopo A., Zappia A., De Bruno A., Pozzo S., Limbo S., Piergiovanni L., Poiana M. (2019). Use of biodegradable materials as alternative packaging of typical Calabrian Provola cheese. Food Packag. Shelf Life.

[27] Peelman N., Ragaert P., Vandemoortele A., Verguldt E., Devlieghere F., De Meulenaer B. Application of biobased materials for packing short, medium and long shelf life food products. Proceedings of the 26th IAPRI symposium on Packaging 2013.

[28] https://thevacuumpouch.co.uk/wp-content/uploads/2021/03/VPC-Eco-Pouch-Spec-Sheet-50mu.pdf.

[29] Panseri S., Martino P.A., Cagnardi P., Celano G., Tedesco D., Castrica M., Chiesa L.M. (2018). Feasibility of biodegradable based packaging used for red meat storage during shelf-life: A pilot study. Food Chem..

[30] Tripathi N., Katiyar V. (2016). PLA/functionalized-gum arabic based bionanocomposite films for high gas barrier applications. J. Appl. Polym. Sci..

[31] Ingallina C., Sobolev A.P., Circi S., Spano M., Giusti A.M., Mannina L. (2020). New hybrid tomato cultivars: An NMR-based chemical characterization. Appl. Sci..

[32] Sobolev A.P., Brosio E., Gianferri R., Segre A.L. (2005). Metabolic profile of lettuce leaves by high-field NMR spectra. Magn. Reson. Chem..

[33] Schneider T., Butz P., Ludwig H., Tauscher B. (2003). Pressure-induced formation of pyroglutamic acid from glutamine in neutral and alkaline solutions. LWT—Food Sci. Technol..

[34] Liang T., Wei F., Lu Y., Kodani Y., Nakada M., Miyakawa T., Tanokura M. (2015). Comprehensive NMR analysis of compositional changes of black garlic during thermal processing. J. Agric. Food Chem..

[35] Prior R.L., Wu X., Schaich K. (2005). Standardized Methods for the Determination of Antioxidant Capacity and Phenolics in Foods and Dietary Supplements. J. Agric. Food Chem..

[36] Kulczyński B., Gramza-Michałowska A. (2019). The Profile of Secondary Metabolites and Other Bioactive Compounds in Cucurbita pepo L. and Cucurbita moschata Pumpkin Cultivars. Molecules.

[37] Can-Cauich C.A., Sauri-Duch E., Victor M.M., Betancur-Ancona D., Cuevas-Glory L.F. (2019). Effect of extraction method and specie on the content of bioactive compounds and antioxidant activity of pumpkin oil from Yucatan, Mexico. Food Chem..

[38] Świder O., Roszko M.L., WòJcicki M., Szymczyk K. (2020). Biogenic amines and free amino acids in traditional fermented vegetables –dietary risk evaluation. J. Agric. Food Chem..

[39] Sánchez-Pérez S., Comas-Basté O., Rabell-González J., Veciana-Nogués M., Latorre-Moratalla M., Vidal-Carou M. (2018). Biogenic amines in plant-origin foods: Are they frequently underestimated in low-histamine diets?. Foods.

[40] Lavizzari T., Veciana-Nogués M.T., Weingart O., Bover-Cid S., Mariné-Font A., Vidal-Carou M.C. (2007). Occurrence of biogenic amines and polyamines in spinach and changes during storage under refrigeration. J. Agric. Food Chem..

[41] Jay J.M., Golden D.A., Loessner M.J. (2005). Modern Food Microbiology.

[42] Ministere de l’Economie des Finances et du Budget (1988). Marché consommation, Produits vegetaux prets a l’emploi dits de la ‘IVemme Gamme’: Guide de bonnes pratique hygieniques. J. Off. De La Repub. Fr..

[43] Corbo M.R., Altieri C., D’Amato D., Campaniello D., Del Nobile M.A., Sinigaglia M. (2004). Effect of temperature on shelf life and microbial population of lightly processed cactus pear fruit. Postharvest Biol. Technol..

[44] Gutiérrez-López G., Barbosa-Canóvas G., WeltiChanes J., Parada-Arias E. (2008). Use of Tapioca Starch Edible Film Containing Potassium Sorbate to Extend the Shelf Life of Minimally Processed Pumpkin. Food Engineering: Integrated Approaches.

[45] Guzman Norleyn M.N., Orellana L.E., Obregon Quinones L.G. (2018). Shelf Life of a Mixture of Pumpkin Puree (Cucurbita moschata) During Storage at 4 °C. Adv. J. Food Sci. Technol..

[46] Lucera A., Costa C., Mastromatteo M., Conte A., Del Nobile M.A. (2010). Influence of different packaging systems on fresh-cut zucchini (*Cucurbita pepo*). Innov. Food Sci. Emerg. Technol..

[47] Zhou C.L., Mi L., Hu X.Y., Zhu B.H. (2017). Evaluation of three pumpkin species: Correlation with physicochemical, antioxidant properties and classification using SPME-GC–MS and E-nose methods. J. Food Sci. Technol..

[48] Snaar J.E.M., Van As H. (1992). Probing water compartments and membrane permeability in plantcells by ^1^H NMR relaxation measurements. Biophys. J..

[49] Hills B.P., Remigereau B. (1997). NMR studies of changes in subcellular water compartmentation in parenchyma apple tissue during drying and freezing. Int. J. Food Sci. Tech..

[50] Khan M.I.H., Wellard R.M., Nagy S.A., Joardder M.U.H., Karim M.A. (2016). Investigation of bound and free water in plat-based food material using NMR T2 relaxometry. Innov. Food Sci. Emerg..

[51] Proietti N., Adiletta G., Russo P., Buonocore R., Mannina L., Crescitelli V., Capitani D. (2018). Evolution of physicochemical properties of pear during drying by conventional techniques, portable-NMR, and modelling. J. Food Eng..

