# Supplementary material for: Commercial Bio-Packaging to Preserve the Quality and Extend the Shelf-Life of Vegetables: The Case-Study of Pumpkin Samples Studied by a Multimethodological Approach
Source: Foods. 2021 Oct 14;10(10):2440. doi: 10.3390/foods10102440 (PMC8535681; doi:10.3390/foods10102440)
Supplement: Supplementary file 1 [file foods-10-02440-s001.zip › foods-1401193-supplementary.pdf]

Commercial bio-packaging to preserve the quality and extend the shelf-life of vegetables: the case-study of pumpkin samples studied by a multimethodological approach

Giacomo Di Matteo [1 †], Paola Di Matteo [2 †], Matteo Sambucci [2], Jacopo Tirillò [2] ,Anna Maria Giusti [3], Giuliana Vinci [4], Laura Gobbi [4], Sabrina Antonia Prencipe [4], Andrea Salvo [1], Cinzia Ingallina [1], Mattia Spano [1], Anatoly P. Sobolev [5], Noemi Proietti [5], Valeria Di Tullio [5], Paola Russo [2*], Luisa Mannina [1*] Marco Valente [2]

[1] Dipartimento di Chimica e Tecnologie del Farmaco, Laboratorio di Chimica degli Alimenti, Sapienza Università di Roma, Piazzale Aldo Moro 5, 00182 Roma, Italy

[2] Dipartimento di Ingegneria Chimica Materiali Ambiente, Sapienza Università di Roma, Via Eudossiana 18, 00184, Roma, Italy

[3] Dipartimento di Medicina Sperimentale, Sapienza Università di Roma, Viale Regina Elena, 324, 00161, Roma, Italy

[4] Dipartimento di Management, Sapienza Università di Roma, Via del Castro Laurenziano 9, 00161, Roma, Italy [#] Board member of Italian Society of Food Chemistry

[5] Laboratorio di Risonanza Magnetica "Segre-Capitani", Istituto per i Sistemi Biologici, Area della Ricerca di Roma 1, CNR, via Salaria Km 29,300 00015 Monterotondo (Rm) Italy

† These authors gave an equal contribution to this work

* Correspondence: paola.russo@uniroma1.it; phone.: +39-06-44585565 (P.R.); luisa.mannina@uniroma1.it; phone.: +39-06-49913735 (L.M.)

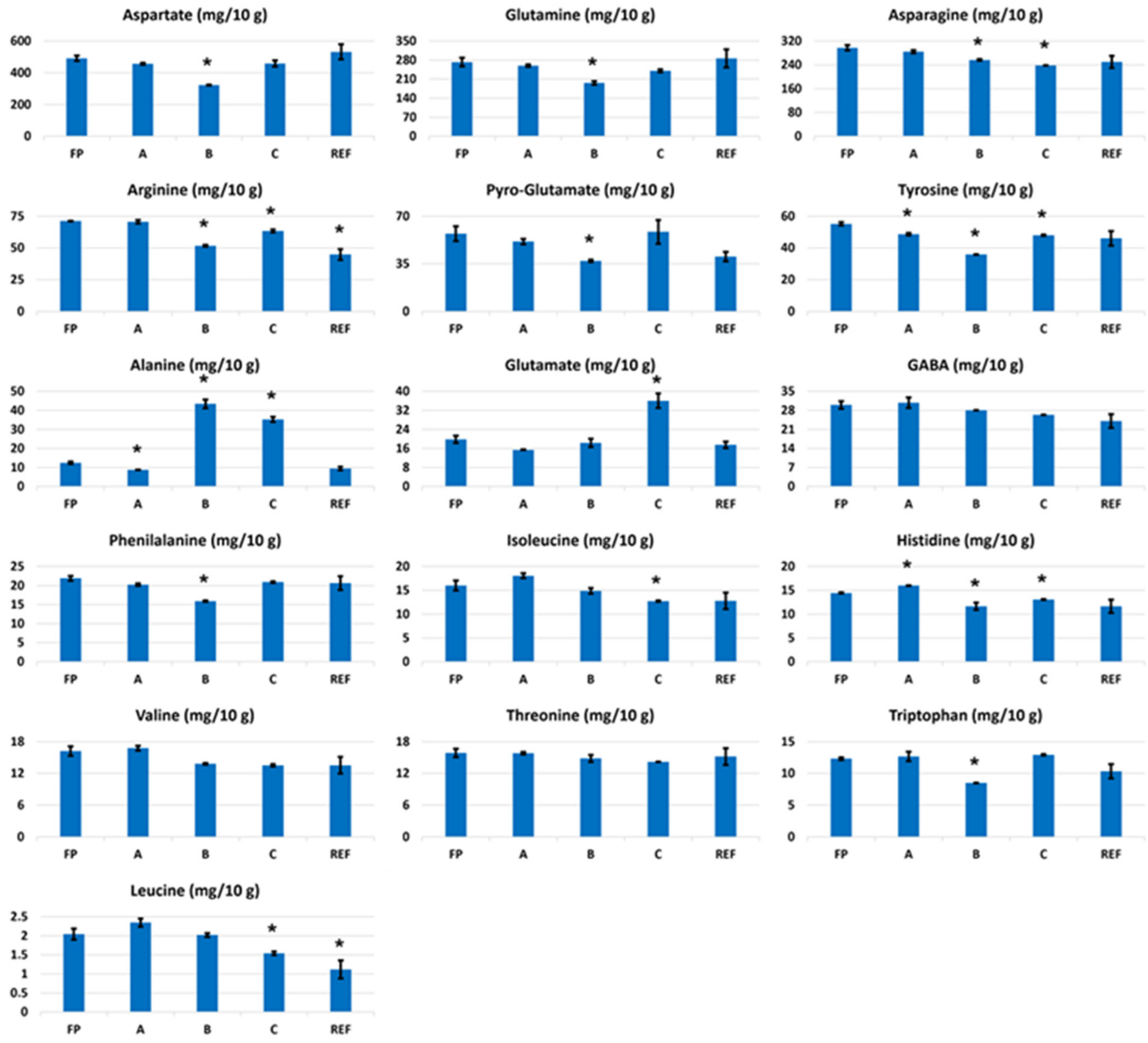

Figure S1. Bar charts of the amino acids identified and quantified (mg/10 g of DW ± SD) in the ^{1}H NMR spectra of hydroalcoholic extracts of freeze dried pumpkins samples. FP=Fresh Pumpkin sample (time 0); A, B and C= commercial bio-films; REF= polyethylene film

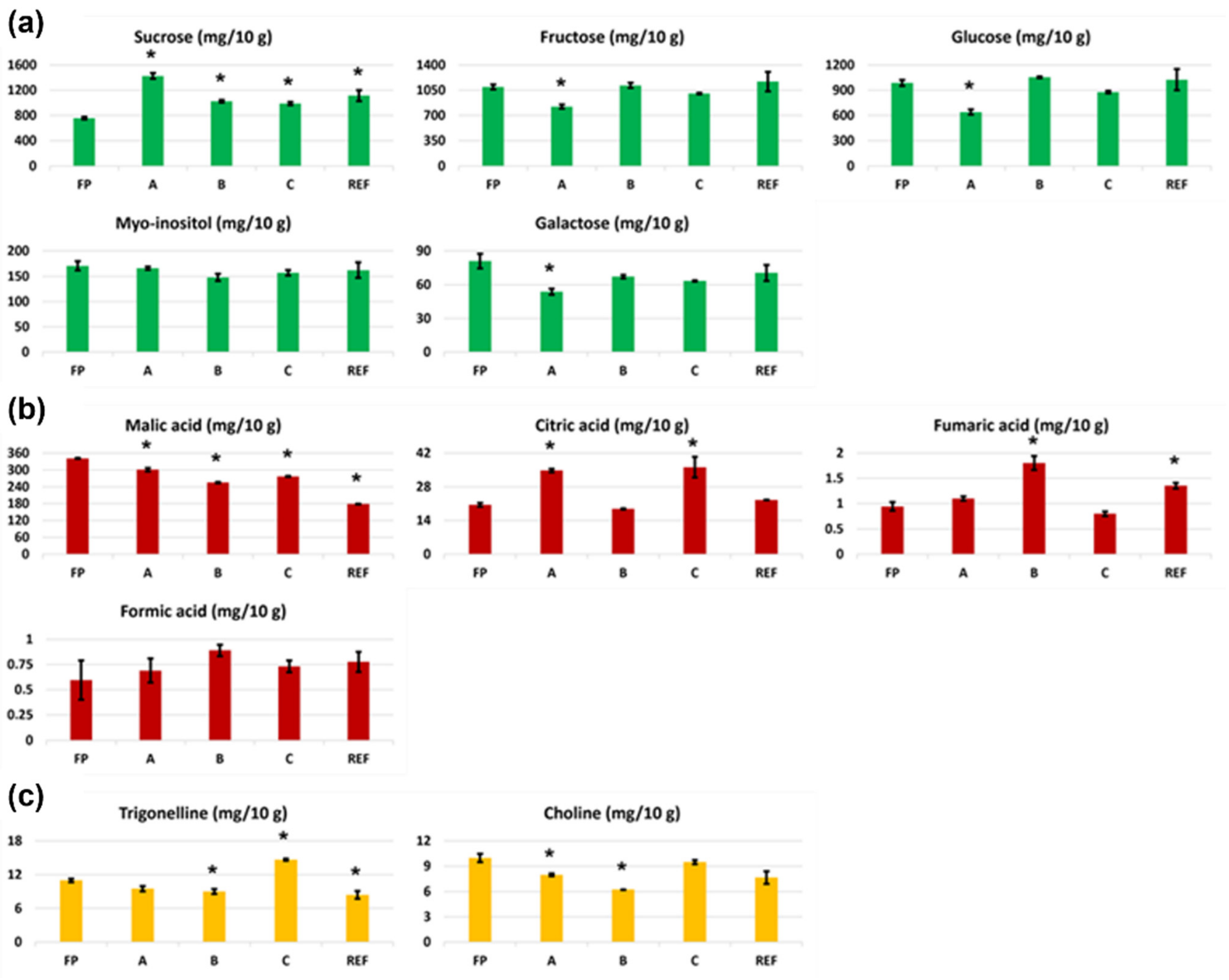

Figure S2. Bar charts of the sugars (**a**), organic acids (b) and other metabolites (**c**) identified and quantified (mg/10 g of DW ± SD) in the ^{1}H NMR spectra of hydroalcoholic extracts of pumpkins. FP=Fresh Pumpkin sample (time 0); A, B and C= commercial bio-films; REF= polyethylene film

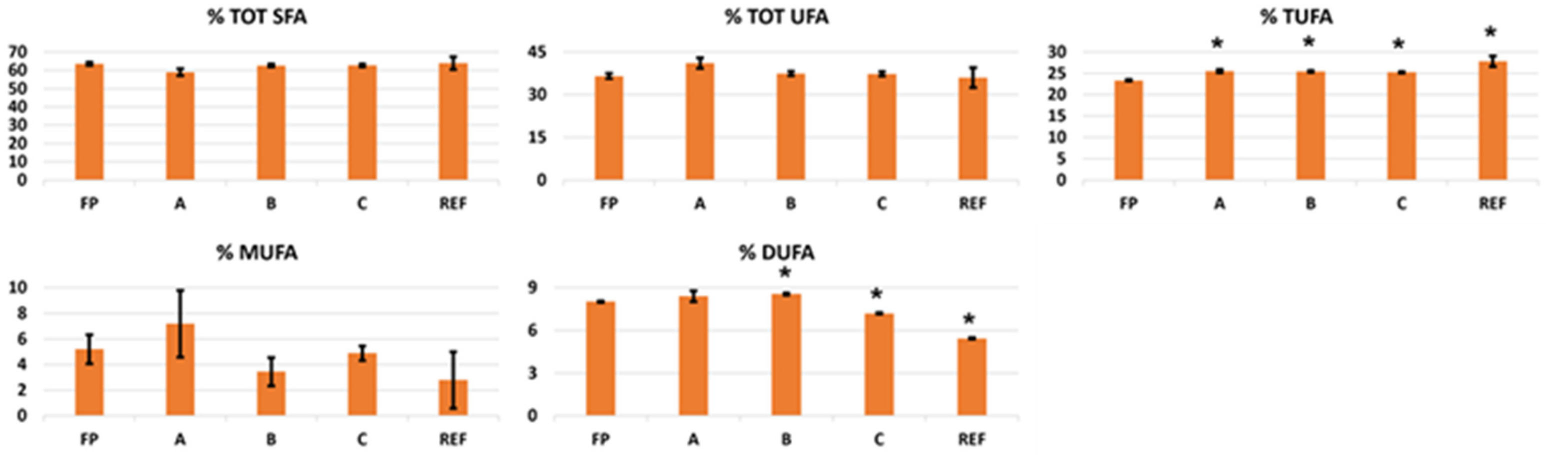

Figure S3. Bar charts of the fatty acids identified and quantified (molar % ± SD) in the ^{1}H NMR spectra of organic extracts of pumpkins. FP=Fresh Pumpkin sample (time 0); A, B and C= commercial biofilms; REF= polyethylene film

Table S1. Metabolites identified in the 600.13 MHz ^{1}H NMR, ^{1}H- ^{1}H TOCSY, ^{1}H-^{13}C HSQC, and ^{1}H-^{13}C HMBC spectra of Bligh–Dyer hydroalcoholic extracts of pumpkins in phosphate buffer/D2O acquired at 28 °C. Asterisks (*) indicate signals selected for integration.

Compound	Assignment	^{1}H (ppm)	Multiplicity [J(Hz)]	^{13}C (ppm)
**Carbohydrates**				
α-D-Fructofuranose	CH-3	4.12*		83.0
	CH-5	4.07		82.4
β-D-Fructofuranose	CH-1,1′	3.60; 3.57		63.8
	CH-3	4.12*		76.4
	CH-4	4.12*		75.4
	CH-5	3.83		81.6
	CH-6,6′	3.81; 3.68		63.3
β-D-Fructopyranose	CH-1,1′	3.72; 3.56		64.8
	CH-3	3.81		68.5
	CH-4	3.90		70.6

	CH-5	4.01		70.2
	CH_2-6,6'	3.71; 4.03		64.4
α-Galactose	CH-1	5.28*	d [3.8]	93.3
	CH-2	3.81		69.3
	CH-3	3.86		70.2
	CH-4	4.00		70.2
	CH-5	4.09		71.5
β-Galactose	CH-1	4.60*	d [7.9]	97.5
	CH-2	3.50		72.9
	CH-3	3.66		73.7
	CH-4	3.94		69.7
	CH-5	3.71		76.1
	CH_2-6	3.74		62.2
α-Glucose	CH-1	5.25*	d [3.8]	93.1
	CH-2	3.55		72.2
	CH-3	3.72		73.7
	CH-4	3.42		70.6
	CH-5	3.83		72.5
	CH_2-6,6'	3.83; 3.78		61.6
β-Glucose	CH-1	4.66*	d [8.0]	97.0
	CH-2	3.26		75.2
	CH-3	3.50		76.8
	CH-4	3.41		70.6
	CH-5	3.47		76.9
	CH_2-6,6'	3.90; 3.74		61.8
Myo-Inositol	CH-1	4.07		73.2
	CH-2,5	3.55		72.4
	CH-3,6	3.63		73.5
	CH-4	3.29*	t [9.4]	75.4

Sucrose	CH-1 (Glucose)	5.42*	d [3.8]	93.2
	CH-2	3.57		72.1
	CH-3	3.77		73.5
	CH-4	3.48		70.2
	CH-5	3.85		73.5
	CH_2-6	3.82		61.1
	CH-1′ (Fructose)	3.69		62.4
	CH-3'	4.22	d [8.8]	77.5
	CH-4'	4.06		75.1
	CH-5'	3.90		82.4
	CH_2-6'	3.82		63.4
**Organic acids**				
Citric acid	α,γ-CH	2.54*	d [15.5]	46.4
	α',γ'-CH	2.68		46.4
	β-C			76.4
Formic acid	HCOOH	8.47*	s	
Fumaric Acid	α,β–CH=CH	6.53*	s	
Malic acid	α-CH	4.30*	dd [9.9; 3.2]	71.3
	β,β'-CH_2	2.39; 2.70	dd [15.3; 9.9]; dd [15.3; 3.2]	43.6
	C			182.6
**Amino acids**				
Alanine	α-CH	3.79		51.5
	β-CH_3	1.49*	d [7.3]	17.2
	COOH			177.0
Arginine	α-CH	3.78		55.1
	β-CH_2	1.93	m	28.6
	γ,γ′-CH_2	1.66; 1.73*	m	24.9

	δ-CH_2	3.24		41.4
Asparagine	α-CH	4.01		52.2
	β,β'-CH_2	2.88*; 2.96	dd [7.2; 16.9]; dd[4.5; 16.9]	35.7
	COOH			175.4
Aspartate	α-CH	3.90		53.1
	β,β'-CH_2	2.71; 2.81*	dd [8.1; 17.5]; dd[3.8; 17.5]	37.5
	γ-COO-			178.5
	COOH			175.3
γ-Aminobutyrate	α-CH_2	2.30*	t [7.4]	35.4
	β-CH_2	1.91		24.7
	γ-CH_2	3.02	t [7.7]	40.4
				182.7
Glutamine	α-CH	3.78		55.1
	β,β'-CH_2	2.15	m	27.4
	γ-CH	2.46*	m	31.9
	γ–CO			178.6
	COOH			175.0
Glutamate	α-CH	3.77		55.7
	β,β'-CH_2	2.07; 2.12	m	28.0
	γ-CH_2	2.35*	m	34.4
	δ–COOH			181.2
Histidine	CH-2	8.12*	d [1.0]	
	CH-4	7.18		118.3
	α-CH	4.01		55.4
	β,β'-CH_2	3.24; 3.30		28.2
Isoleucine	α-CH	3.68		60.5
	β-CH	1.98		36.8
	γ,γ'-CH_2	1.27; 1.47		25.5

	γ-CH_3	1.01	d [7.1]	15.7
	δ-CH_3	0.94*	t [7.6]	12.1
Leucine	α-CH	3.73		54.3
	β-CH_2	1.73		40.8
	γ-CH	1.70		25.2
	δ-CH_3	0.97*	d [6.1]	23.0
	δ'-CH_3	0.96	d [6.2]	22.0
Phenylalanine	α-CH	4.00		56.9
	β,β'-CH_2	3.16; 3.27		37.4
	CH-2,6	7.34	dd [8.1; 1.2]	130.5
	CH-4	7.39	tt [7.3; 1.2]	128.6
	CH-3,5	7.43*	t [7.8]	130.2
	C1			136.2
Pyro-Glutamate	α-CH	4.18*	dd [5.9; 9.1]	59.3
	β,β'-CH_2	2.04; 2.51	m	26.5
	γ-CH_2	2.41	m	30.7
	CO			182.8
	COOH			181.4
Threonine	α-CH	3.60		61.4
	β-CH	4.26		67.0
	γ-CH_3	1.34*	d [6.6]	20.5
Tryptophan	α-CH	4.07		55.9
	β,β'-CH_2	3.33; 3.48		27.4
	CH-4	7.28	t [7.6]	123.1
	CH-5	7.20	m	120.4
	CH-6	7.74	d [8.0]	119.5
	CH-7	7.55*	d [8.1]	113.0
Tyrosine	α-CH	3.95		57.1
	β,β'-CH_2	3.07; 3.19		36.5

	CH-2,6	7.20	d [8.5]	131.7
	CH-3,5	6.91*	d [8.5]	116.9
Valine	α-CH	3.62		61.3
	β-CH	2.28		30.2
	γ-CH_3	1.00	d [7.0]	17.7
	γ'-CH_3	1.05*	d [7.0]	19.0
**Miscellaneous metabolites**				
Choline	$N(CH_3)_3+$	3.21*	s	54.9
	α-CH_2	3.52		68.4
	β-CH_2-OH	4.06		56.6
Trigonelline	CH_3	4.45	s	49.2
	CH-1	9.12	s	
	CH-3,5	8.84*	d [7.0]	
	CH-4	8.10	t [7.0]	128.7

Table S2. Metabolites identified in the 600.13 MHz ^{1}H NMR, ^{1}H- ^{1}H TOCSY, ^{1}H-^{13}C HSQC, and ^{1}H-^{13}C HMBC spectra of Bligh–Dyer organic extracts of pumpkins $CDCl_3$/CD_3OD (2:1 v/v) mixture acquired at 28 °C. Asterisks (*) indicate signals selected for integration. For the integration of total fatty acids (I_{FA}) and total unsaturated fatty acids (I_{UFA}), the region of 2.20–2.36 ppm and 5.25–5.40 ppm, respectively, were considered.

Compound	Assignment[a]	^{1}H (ppm)	Multiplicity: *J* [Hz]	^{13}C (ppm)
Oleic fatty chain (C18:1 Δ^9)	COO			174.1
	CH_2-2	2.30		34.2
	CH_2-3	1.57	m	24.9
	CH_2-4,7	1.30	m	29.6
	CH_2-8	2.02	m	27.3
	CH=CH 9,10	5.32	m	130.5
	CH_2-11	2.02	m	27.3
	CH_2-12,16	1.32	m	29.4
	CH_2-17	1.23	m	23.0
	CH_3-18	0.85	t	13.9

Linoleic fatty chain	COO			174.1
(C18:2 $\Delta^{9,12}$)	CH_2-2	2.30		34.2
	CH_2-3	1.57	m	24.9
	CH_2-4,7	1.30	m	29.6
	CH_2-8	2.02	m	27.3
	CH= 9	5.32	m	130.2
	CH= 10	5.31	m	128.3
	CH_2-11	2.73*	t [6.8]	25.6
	CH= 12	5.31	m	128.3
	CH= 13	5.32	m	130.2
	CH_2-14	2.02	m	27.3
	CH_2-15	1.30	m	29.6
	CH_2-16	1.30	m	30.4
	CH_2-17	1.23	m	23.0
	CH_3-18	0.84	t [7.1]	13.9
Linolenic fatty chain	COO			174.1
(C18:3 $\Delta^{9,12,15}$)	CH_2-2	2.30		34.2
	CH_2-3	1.57	m	24.9
	CH_2-4,7	1.30	m	29.6
	CH_2-8	2.03	m	27.3
	CH= 9	5.32	m	130.2
	CH= 10	5.31	m	128.3
	CH_2 11	2.77*	t [6.2]	25.6
	CH=CH 12,13	5.31	m	128.3
	CH_2-14	2.77*	t [6.2]	25.6
	CH= 15	5.29	m	127.9
	CH= 16	5.34	m	132.0
	CH_2-17	2.04	m	20.6
	CH_3-18	0.94	t [7.6]	14.0
Saturated fatty acids	COO			174.1
	CH_2-2	2.30		34.2
	CH_2-3	1.57	m	24.9

CH_2	1.32	m	29.4
CH_2 n-1	1.23		23.0
CH_3 n	0.85	t	13.9

Table S3. One-way analysis of variance (ANOVA) of the resulted NMR quantification of the polar metabolites. F and p values for the different examined packaging that reach a $p < 0.05$ respect to the FP were showed.

	**A**		**B**		**C**		**REF**	
	**F**	***p***	**F**	***p***	**F**	***p***	**F**	***p***
Ile					19.64	0.0473		
Leu					22.16	0.0423	22.07	0.0424
Val								
Thr								
Ala	78.37	0.0125	336.64	0.0030	479.57	0.0021		
Arg			1163.06	0.0009	88.27	0.0111	74.79	0.0131
GABA								
Glu					45.41	0.0213		
Gln			39.95	0.0241				
Citric	365.04	0.0027			25.96	0.0364		
Asp			193.96	0.0051				
Asn			37.31	0.0258	85.93	0.0114		
Chl	31.03	0.0307	117.28	0.0084				
Mio-inositol								
Fructose	76.31	0.0129						
Pyro-Glu			26.56	0.0356				
Malic	84.06	0.0117	2150.62	0.0005	2077.50	0.0005	16878.34	0.0001
Sucrose	364.12	0.0027	168.23	0.0059	91.77	0.0107	31.32	0.0305
Fumaric			55.49	0.0175			31.04	0.0307
Tyr	54.66	0.0178	682.72	0.0015	82.71	0.0119		
Phe			169.27	0.0059				
Trp			688.64	0.0014				
His	278.19	0.0036	26.80	0.0353	180.78	0.0055		
Formic								
Trigonelline			28.22	0.0337	215.67	0.0046	24.39	0.0386

Glucose	104.25	0.0095
Galactose	30.37	0.0314

Table S4. One-way analysis of variance (ANOVA) of the resulted NMR quantification of the fatty acids. F and *p* values for the different examined packaging that reach a *p* < 0.05 respect to the FP were showed.

	**A**		**B**		**C**		**Control**	
	**F**	***p***	**F**	***p***	**F**	***p***	**F**	***p***
DUFA			81.44	0.0121	286.23	0.0035	3187.09	0.0003
TUFA	57.94	0.0168	99.81	0.0099	125.84	0.0079	25.16	0.0376
Tot UFA								
MUFA								
Tot SFA								
